# Supplementary material for: Association between shell morphology of micro-land snails (genus Plectostoma) and their predator’s predatory behaviour
Source: PeerJ. 2014 Apr 1;2:e329. doi: 10.7717/peerj.329 (PMC3976122; doi:10.7717/peerj.329)
Supplement: File S1 [file peerj-02-329-s001.pdf]

1. **Field observation and laboratory observation**

We compiled all the data regarding the interaction between *Plectostoma* and its predators from our field observations conducted between October 2002 and January 2013 in Peninsular Malaysia and Sabah. Most of these observations were made during the day time. Whenever possible, field notes and photographs were taken when interactions between *Plectostoma* species and their predators were seen.

We made five direct observations on the interactions between *Plectostoma* snails and their predators (Table S1). We found two *Pteroptyx* species larvae (Lampyridae) and an *Atopos* slug species (Rathouisiidae) attacking three *Plectostoma* species. *Pteroptyx* was seen to attack adult and juvenile *Plectostoma* snails by shell-apertural entry whereas *Atopos* were seen to attack adult *Plectostoma* snails by shell-drilling.

Table S1. Observation of predators and their predatory behaviour towards *Plectostoma* species in the field.

No. of occasion	Date and time	Location	Note	Observer
1	28th March 2003, Probably between 09:00 – 10:00 PM.	Malaysia, Sabah, Tomanggong Besar.	*Atopos* slug attacked *Plectostoma fraternum* (Schilthuizen et al. 2006). A total of 15 slugs were found within 25 m^2 og limestone rock face.	Menno Schilthuizen
2	9th May 2011, 11:30 AM	Malaysia, Sabah, Gomantong Cave.	*Pteroptyx tener* larva attacked *Plectostoma concinnum* (shell-apertural entry).	Liew Thor-Seng
3	9th May 2011, 11:34 AM	Malaysia, Sabah, Gomantong Cave.	*Pteroptyx tener* larva attacked *Plectostoma mirabile* (shell-apertural entry).	Liew Thor-Seng
4	28th May 2011, 10:25 AM	Malaysia, Kelantan, Kampung Bayu.	*Pteroptyx* cf. *valida* larva attacked *Plectostoma laidlawi* (shell-apertural entry).	Liew Thor-Seng
5	14th December 2011, 10:00 AM	Malaysia, Sabah, Batu Kampung.	*Atopos* slug attacked *Plectostoma concinnum* (shell-drilling).	Liew Thor-Seng & Mohd. Effendi Marzuki
6	20th January 2013, 09:15 AM	Malaysia, Sabah, Batu Kampung.	*Atopos* slug attacked *Plectostoma concinnum* (shell-drilling).	Liew Thor-Seng
7	18th January 2013, 10:30 AM	Malaysia, Sabah, Batu Kampung.	*Atopos* slug.	Liew Thor-Seng
8	21st January 2013, 08:25 AM	Malaysia, Sabah, Batu Kampung.	*Atopos* slug.	Liew Thor-Seng
9	18th January 2013, 10:15 AM	Malaysia, Sabah, Batu Kampung.	*Pteroptyx tener* larva.	Liew Thor-Seng
10	20th January 2013, 08:40 AM	Malaysia, Sabah, Batu Kampung.	*Pteroptyx tener* larva.	Liew Thor-Seng

2. Observation of drill hole characteristic.

***Atopos* drill hole characteristics on the shell of adult *Plectostoma*.**

An *Atopos* slug with a body length of 14 mm, was collected from the rock face of Batu Kampung (5° 32’11”N, 118°12’47”E, hereafter Site A) (Figure 1C, No. 5 in Table S1). At the same time, 250 living adult and juvenile *P. concinnum* were collected from the same location. After that, the *Atopos* and the *P. concinnum* snails were kept in a tank (30 cm × 30 cm × 14 cm). The micro-habitat in the tank was set up to mimic the natural habitat at site A, and consisted of limestone rock pieces and temperature (25°C - 30°C) and humidity (95% - 100%) control. During the test, which lasted from 19th December 2011 to 24th February 2012, we regularly collected empty shells of dead *Plectostoma* from the tank. Adult empty shells with drill holes were retained for analysis. Empty shells without drill holes were discarded as the cause of death cannot be ascertained. The test ended when the *Atopos* was no longer seen, and presumably dead. The diameter and position of drill holes on the shells were examined and the number of ribs of each shell was quantified.

We found drill holes made by an *Atopos* slug, in six empty *Plectostoma concinnum* shells (Supplementary materials File S2, Page 1: Table S1, Figure S1). The experimental slug did not show any stereotyped choice of drill location on the shells. As shown in Figures 1E and 1F (see main text), these drill holes are distinctive with a narrow scraped rim around the margin. All the drill holes that were made by the same slug had uniform size (mean diameter = 0.21 mm, SD = 0.01 mm, n = 6). Of these six prey shells, two had the drill hole located in between two ribs and four had the drill hole through the ribs. The numbers of ribs of the six shells vary from 93 to 108 (mean = 98, SD = 6, n = 6).

3. **Literature survey of behaviour of Lampyridae beetle larvae and Rathouisiidae slugs towards land snails**

In addition to the field observations, we compiled published literature on the predatory behaviour towards land snails for the two predators that were identified from our field observations, namely Rathouisiidae slugs and Lampyridae beetle larvae. We used the search engines of Web of Science and Google Scholar on 23rd May 2013, with the keywords (rathouis* AND snail*) and (lampyrid* AND snail*).

We could not find any literature regarding to the predatory behaviour of the species *Pteroptyx* cf. *valida* and *Pteroptyx tener* on land snails. Nevertheless, beetle larvae of other genera in Lampyridae were recorded to attack land snails varying in size (the smallest being 2 mm) by shell-apertural entry (Table S2), in which the larva inserts its elongate head into the shell via the shell aperture.

Published information on the *Atopos* slug's predatory behaviour towards land snails was similarly scarce (Table S3). Despite this, other genera in the Rathouisiidae are reported to use two different predatory strategies to attack land snails, namely, shell-apertural entry and shell-drilling (Table S3).

Table S2. Literature survey of predatory behaviours of Lampyridae beetle larvae towards land snails.

Source	Predator	Habitat	Predatory strategy	Note
Madruga Rios and Hernández Quinta (2010)	*Alecton discoidalis* Laporte, 1833	Limestone forest, on left litter and on rock.	Shell–apertural entry.	- Preyed on 13 land snails species of 7 families.<br>- Was found associated with operculate gastropods.
Clench and Jacobson (1968)	*Alecton* sp.	Limestone.	Shell–apertural entry.	- Land snail species *Viana regina* (Helicinidae).
Wang et al. (2007)	*Pyrocoelia pectoralis* (E. Oliv., 1883)	Grasslands and deserted farmlands.	Shell–apertural entry.	- Inserted their elongate heads together with their mouthparts into the shell to bite and chew at the snail bodies.
Archangelsky and Branham (1998)	*Pyractomena borealis* (Randall, 1838)	n.a.	Shell–apertural entry.	- Inserted their elongate heads together with their mouthparts into the shell to eat the content of retracted snail.<br>- Injected extraoral disgesting fluids through their mandibles.
Thornton (1997:65)	lampyrid	Leaf litter.	n.a.	- Preyed on very small snail (2 mm), possibly *Gastrocopta pediculus*.

Table S3. Literature survey of predatory behaviours of Rathouisiidae slugs towards land snails.

Source	Predator	Habitat	Predatory strategy	Note
Heude (1882-1890)	*Rathouisia leonina* Heude, 1882	Wet and shaded brick wall cracks of the building.	Shell–apertural entry.	- Could eat up to two small prey and stay up to three days without food.
Wu et al. (2006)	*Rathouisia leonina* Heude, 1882	Limestone hills and urban garden, humid and shady habitat.	Shell–apertural entry and shell-drilling.	- Inserted its head or its proboscis alone through aperture depending upon aperture size of the prey.<br>- Fed on eggs by drilling a hole.<br>- The size of bored hole is related to the size of the predator.
Tan and Chan (2009)	*Atopos* sp.		Shell–apertural entry.	- Held the prey with the anterior part of the foot arched in a straddling position.<br>- Inserted its proboscis into the prey shell via aperture.
Kurozumi (1985)	*Incillaria* sp.	Around limestone rocks, wet part of the forest.	Shell–apertural entry and shell-drilling.	- Fed on eggs by drilling a hole.<br>- There was a narrow scraped part on the margin of the hole.<br>- Drilled hole on the shell of prosobranch snails such as *Georissa fukudai* and *Cyclophoris turgidus*.<br>- Attacked other snails through aperture.

Reference

Archangelsky M, Branham MA. 1998. Description of the preimaginal stages of *Pyractomena borealis* (Randall, 1838) (Coleoptera: Lampyridae) and notes on its biology. *Entomological Society of Washington* 100:421-430.

Clench WJ, Jacobson MK. 1968. Monograph of the Cuban genus *Viana* (Mollusca: Archaeogastropoda: Helicinidae). *Breviora* 298:1-25.

Heude PM. 1882-1890. Notes sur les mollusques terrestres de la vallee du Fleuve Bleu. *Mémoires concernant l'histoire naturelle de l'Empire Chinois*: 1-179.

Kurozumi T. 1985. Evidence of slug predation on land snail eggs. *Applied Entomology and Zoology* 20(4):490-491.

Tan SK, Chan SY. 2009. New records of predatory slugs from Singapore with notes on their feeding behaviour. *Nature in Singapore* 2:1-7.

Madruga Rios O, Hernández Quinta M. 2010. Larval Feeding Habits of the Cuban Endemic Firefly *Alecton discoidalis* Laporte (Coleoptera: Lampyridae). *Psyche: A Journal of Entomology* 2010:Article ID 149879, 5 pages, doi:10.1155/2010/149879

Thornton IW. 1997. *Krakatau: the destruction and reassembly of an island ecosystem*. Harvard University Press.

Wang Y, Fu X, Lei C, Jeng ML, Nobuyoshi O. 2007. Biological Characteristics of the Terrestrial Firefly *Pyrocoelia pectoralis* (Coleoptera: Lampyridae). *The Coleopterists Bulletin* 61(1):85-93.

Wu M, Guo JY, Wan FH, Qin QL, Wu Q, Wiktor A. 2006. A preliminary study on the biology of the predatory terrestrial mollusk *Rathouisia leonina*. *Veliger* 48(2):61-74.
