# Supplementary material for: Association between shell morphology of micro-land snails (genus Plectostoma) and their predator’s predatory behaviour
Source: PeerJ. 2014 Apr 1;2:e329. doi: 10.7717/peerj.329 (PMC3976122; doi:10.7717/peerj.329)
Supplement: File S2 [file peerj-02-329-s002.pdf]

Test 1 (a) – Slug's predatory behaviour against adult *P. concinnum* (with fully grown shell)

Figure S1. Shells that were preyed by *Atopos* slugs in Test 1(a)

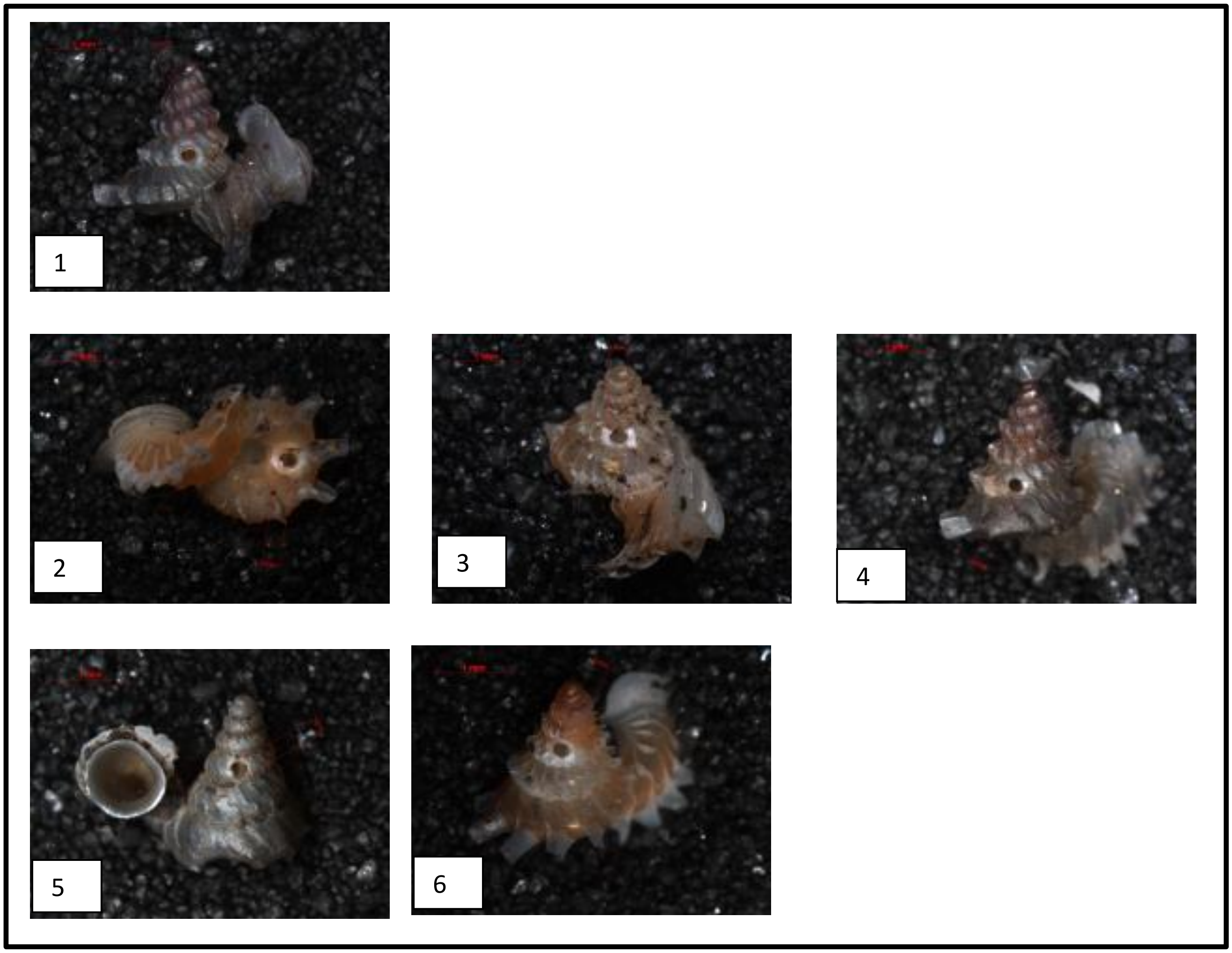

Table S1. Parameters of the shell and drill hole of specimens of Test 1(a)

specimen	Hole diameter	number of ribs on shell	hole on ribs (1) or between two ribs (0)
1	0.19	97	0
2	0.22	108	0
3	0.22	93	1
4	0.19	94	1
5	0.22	94	1
6	0.22	106	1

<u>Test 1 (b) – Association between slug's shell-drilling behaviour, and adult snail's shell tuba and ribs intensity.</u>

Figure S2. Specimens from population T7.

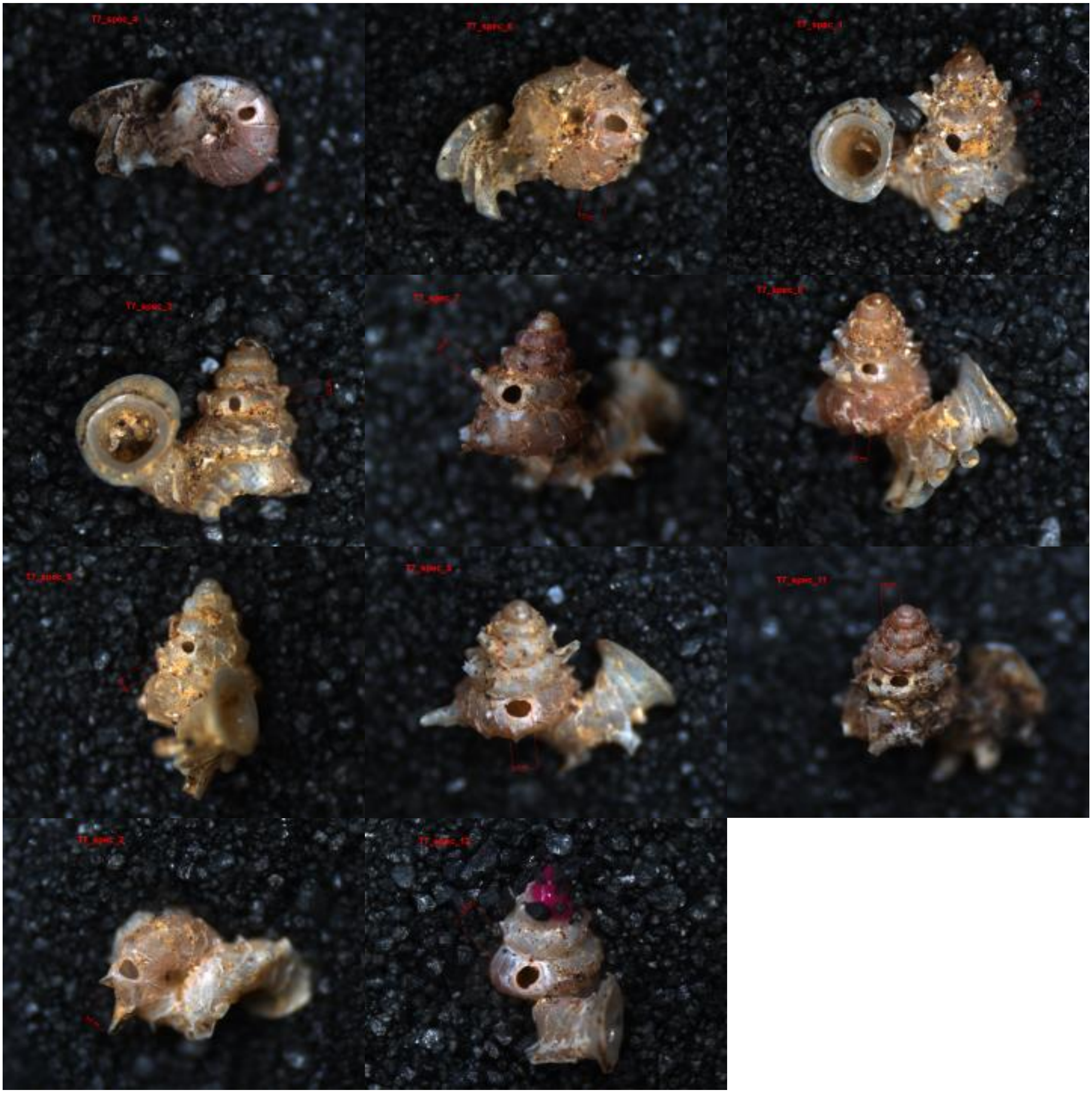

Figure S3. Specimens from population T21.

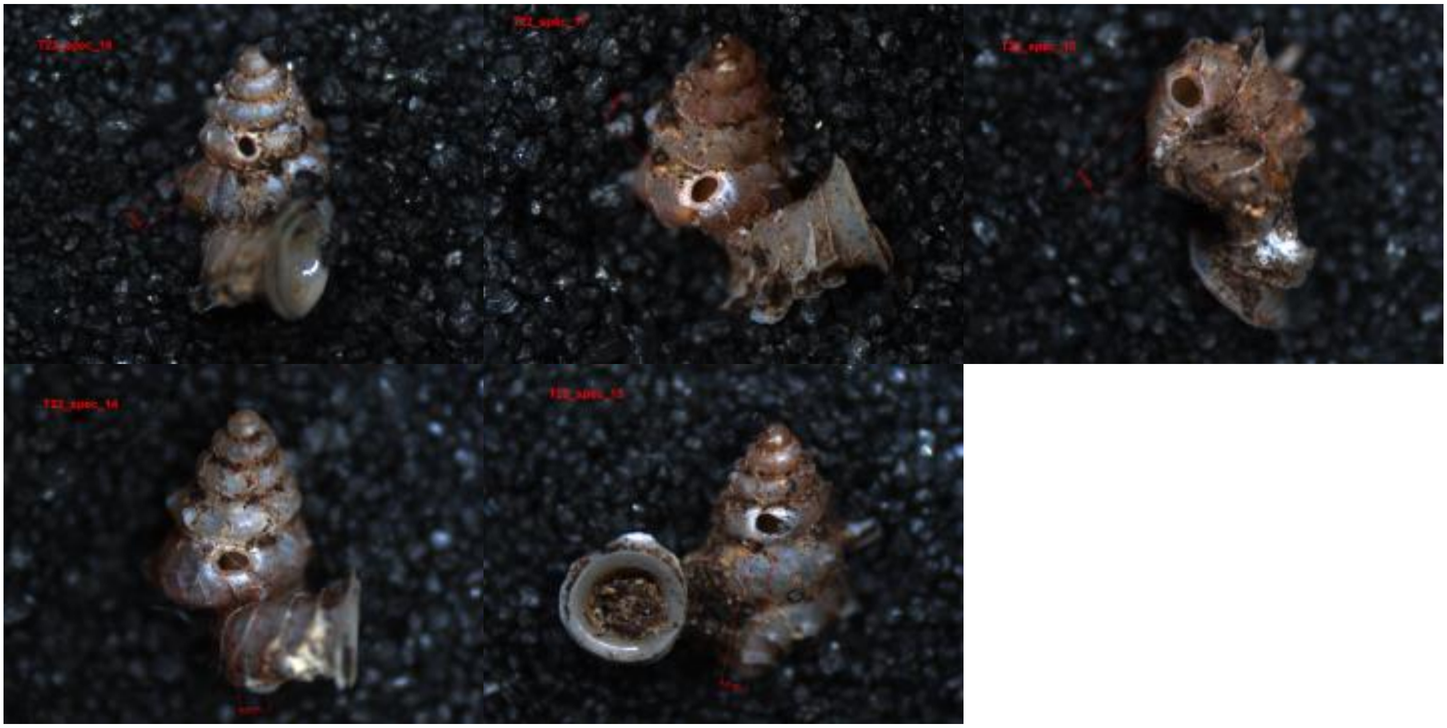

Figure S4. Specimens from population T22.

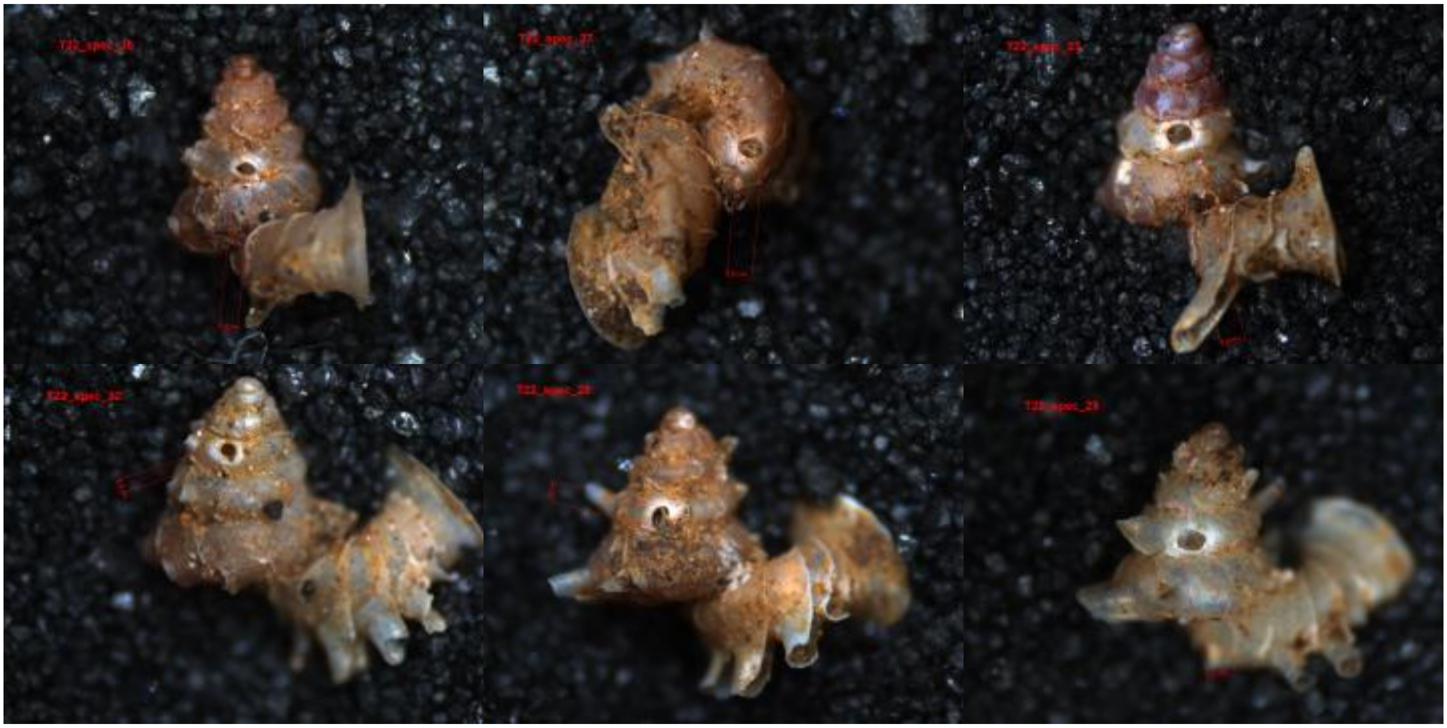

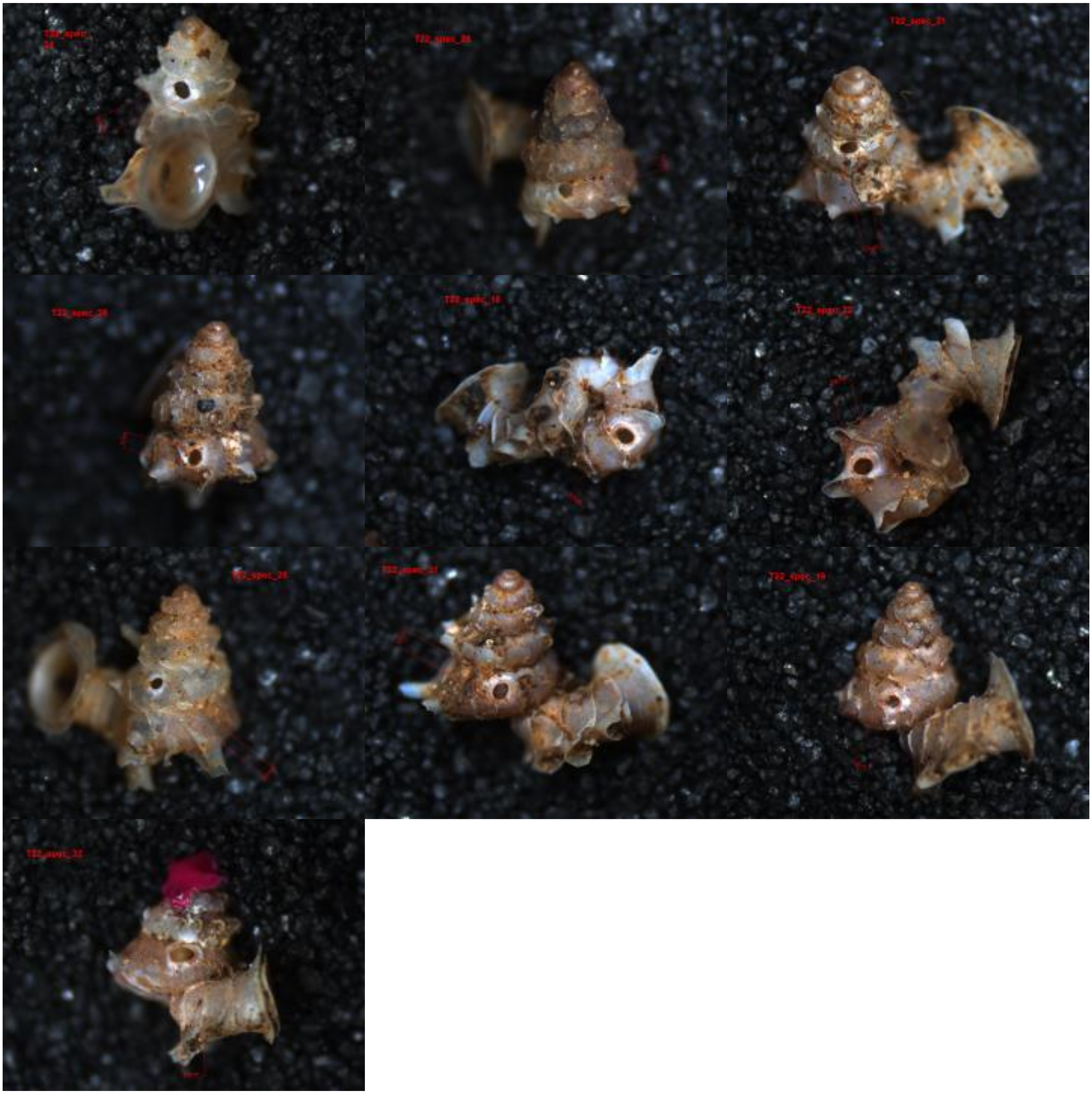

Figure S5. Specimens from population T 45.

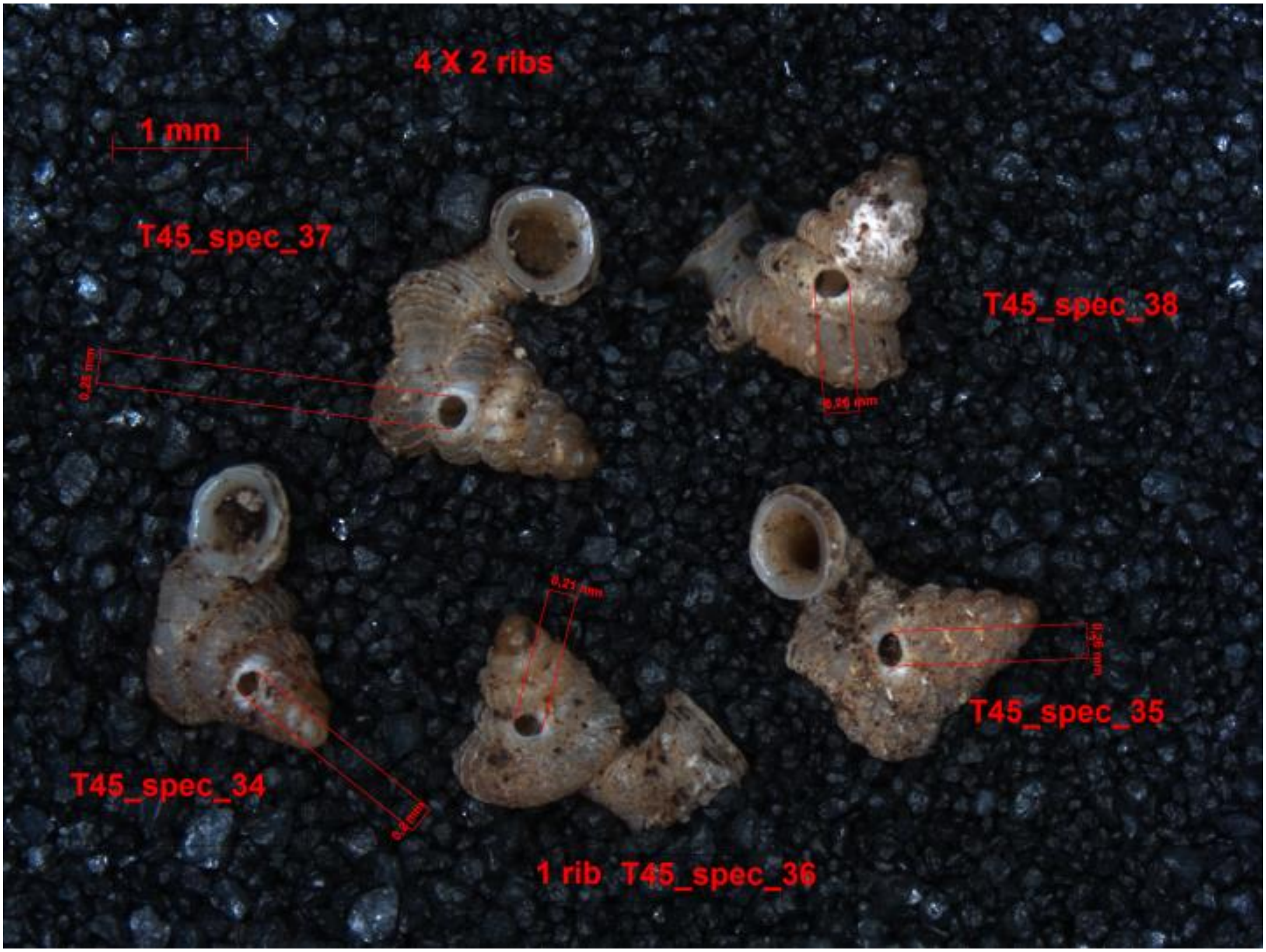

Figure S6. Specimens from population BOR 2169.

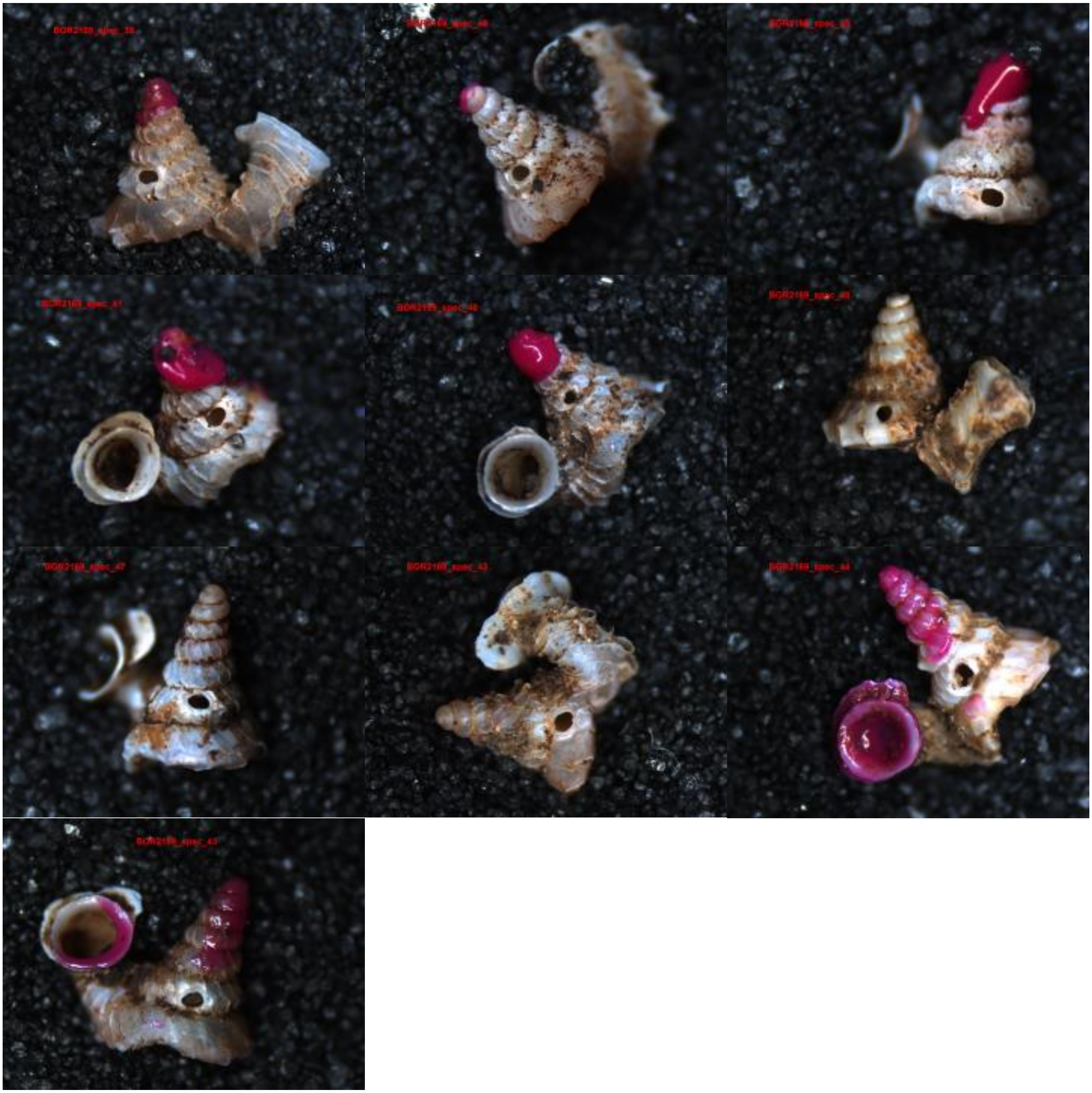

Figure S7. Specimens from population BOR 1690.

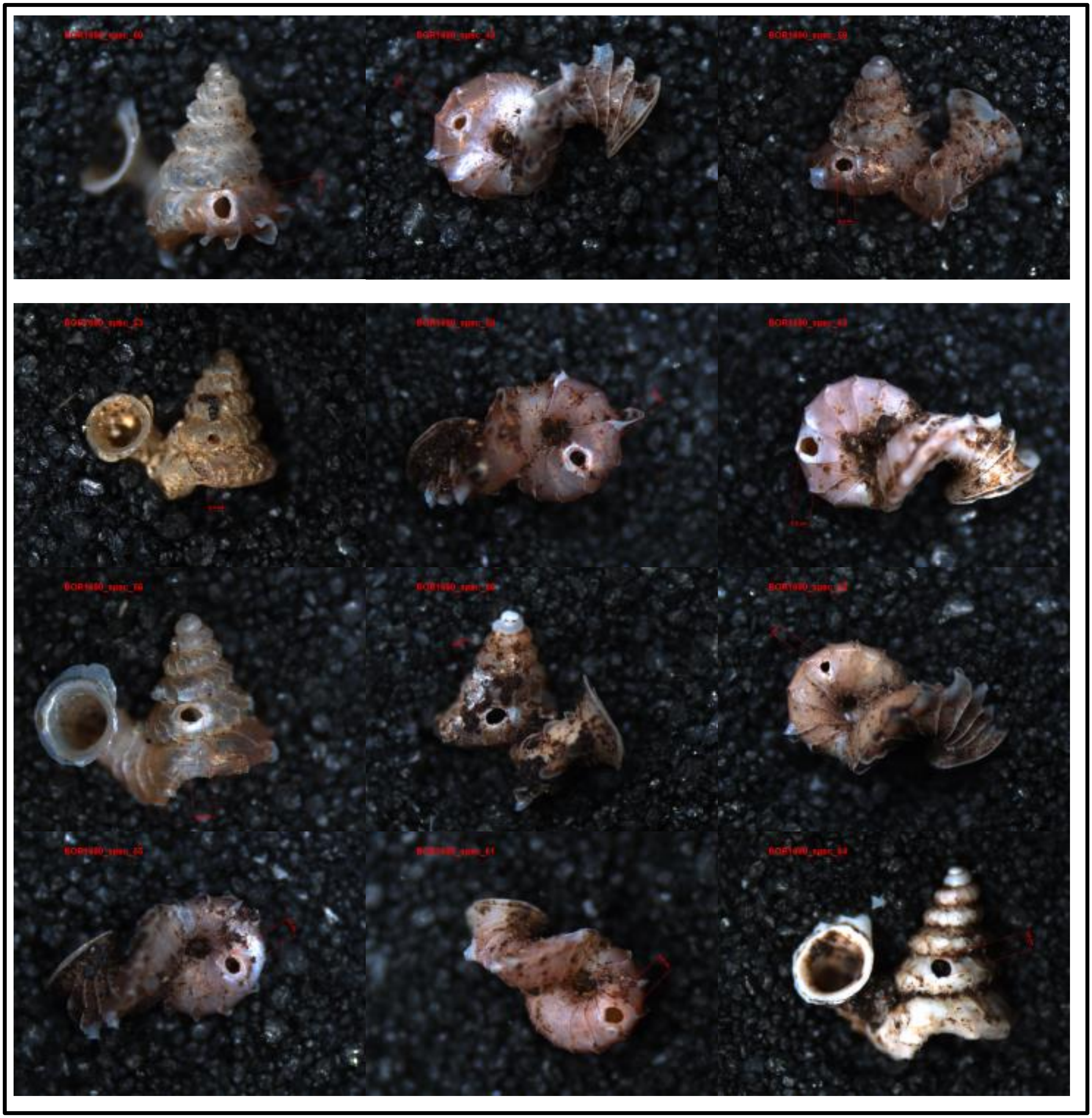

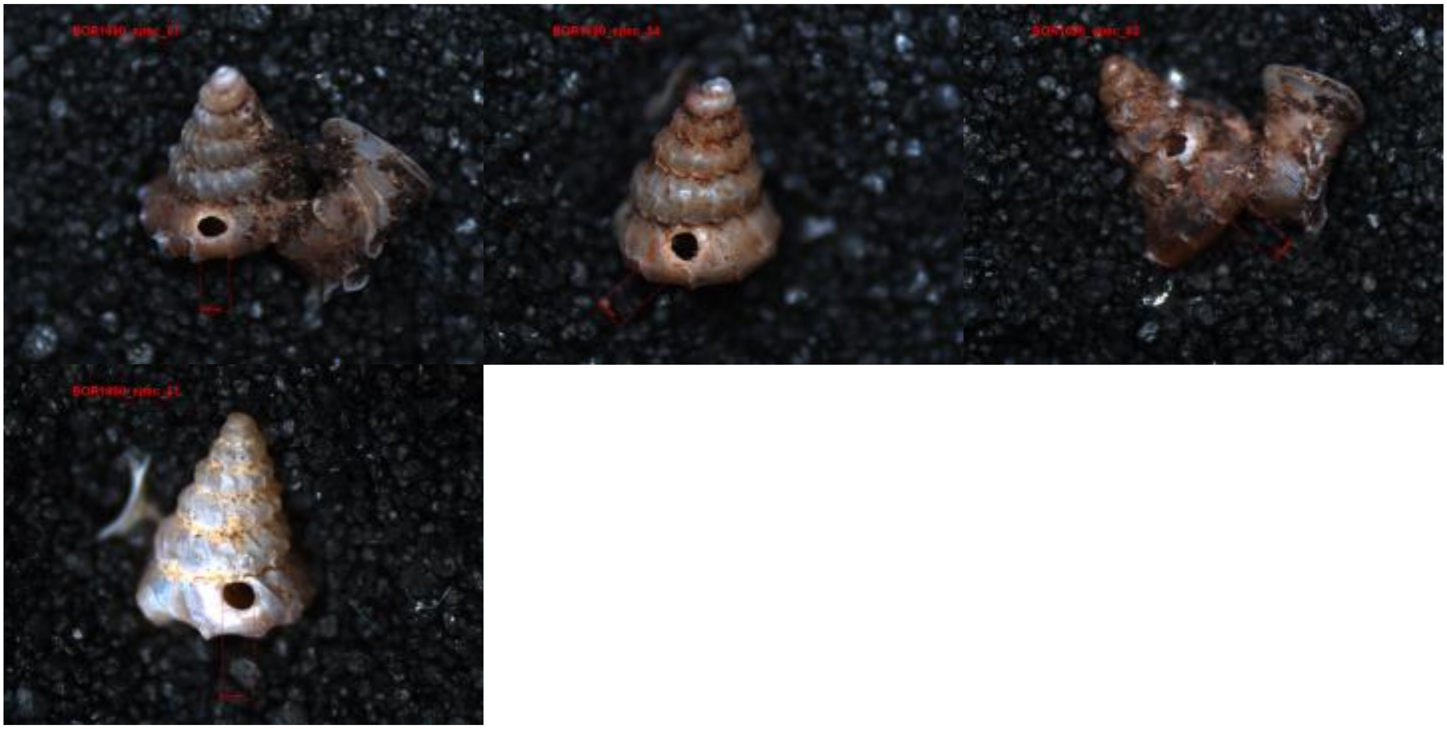

Figure S8. Specimens from population T 29.

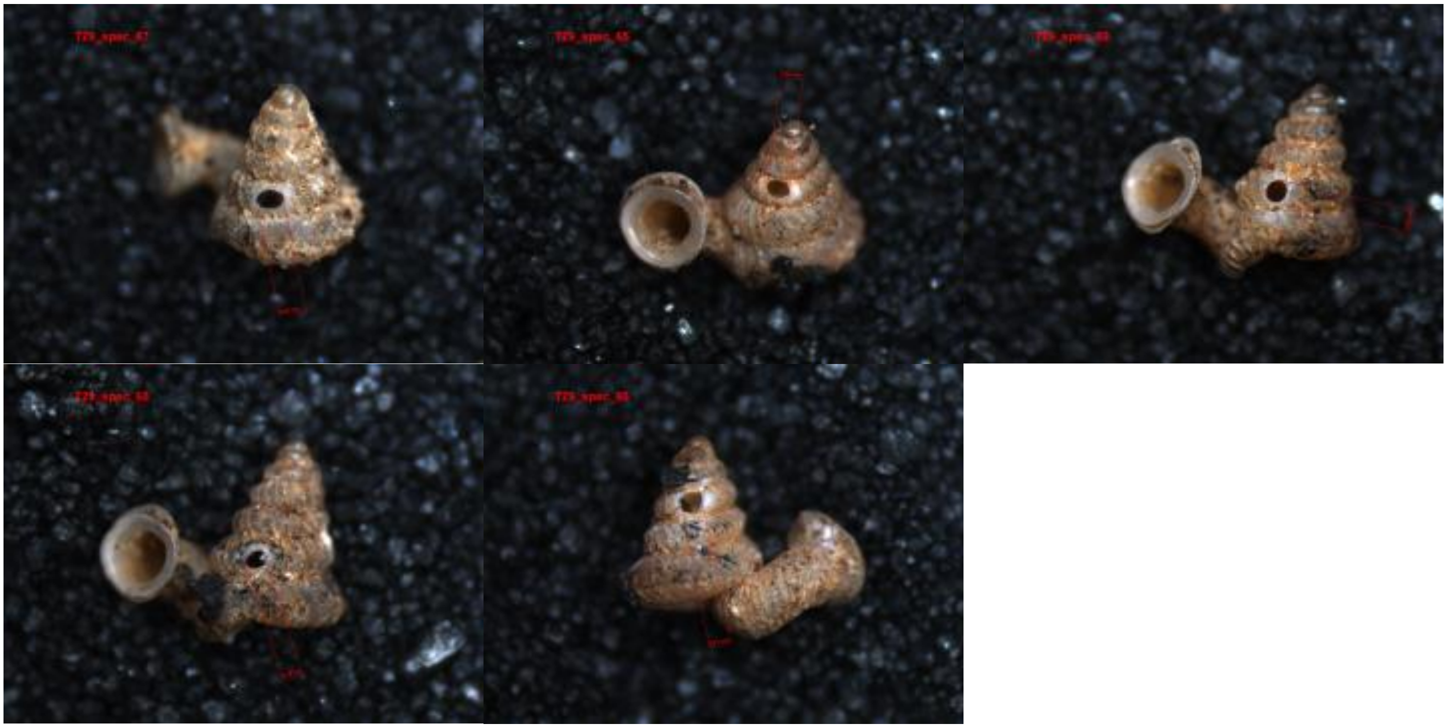

Figure S9. Specimens from population T 33

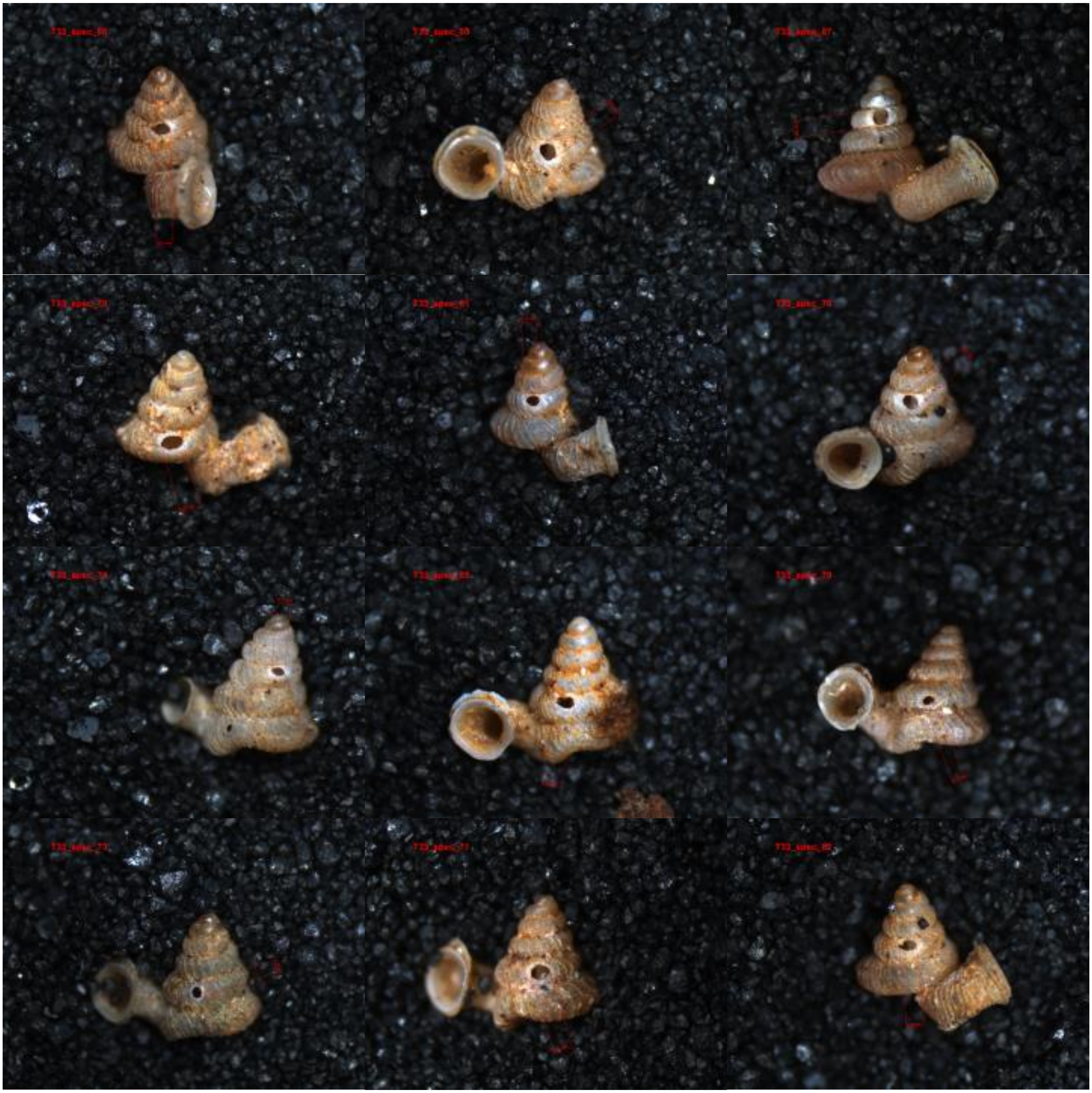

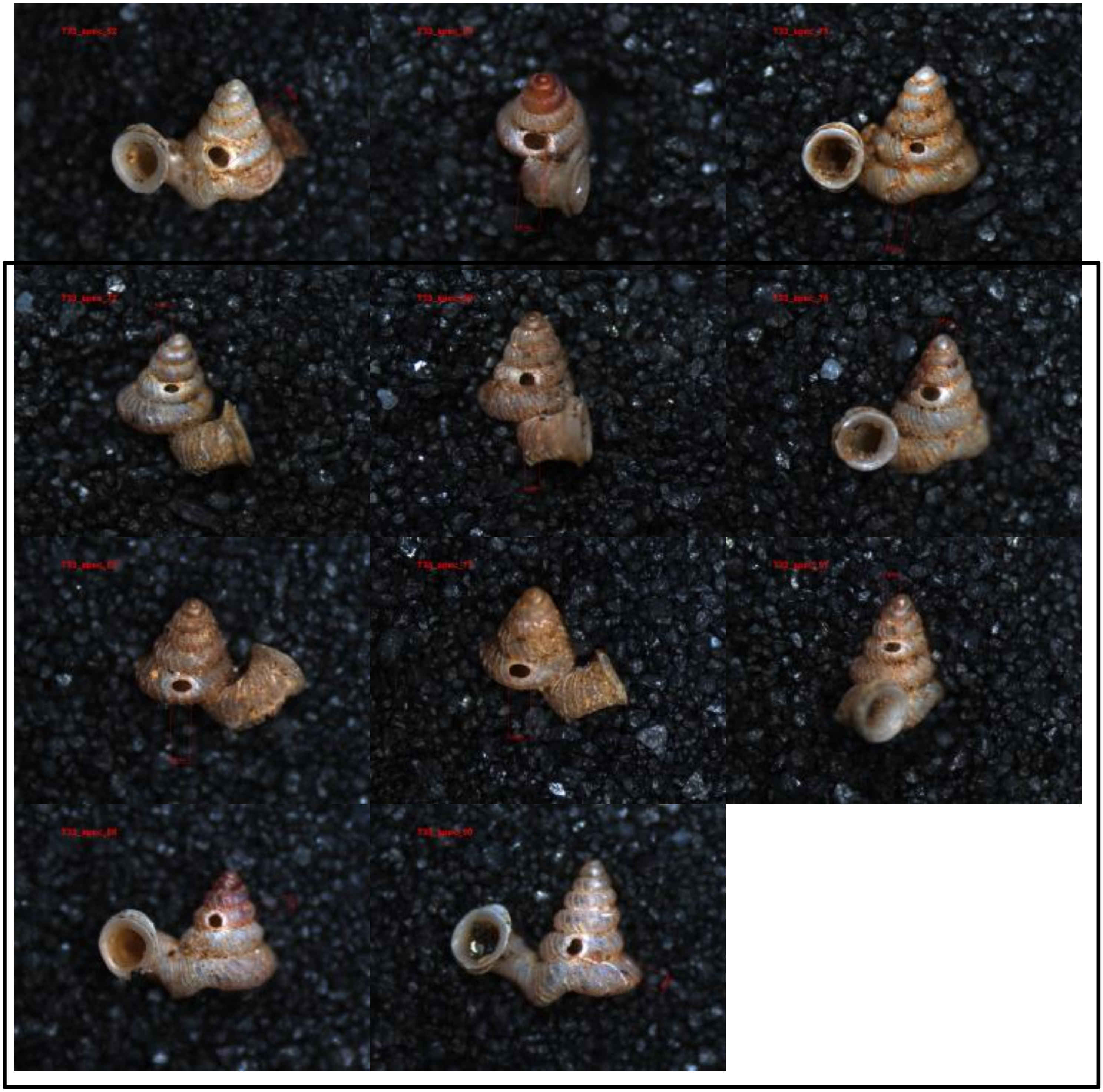

Figure S10. Specimens from population T 42.

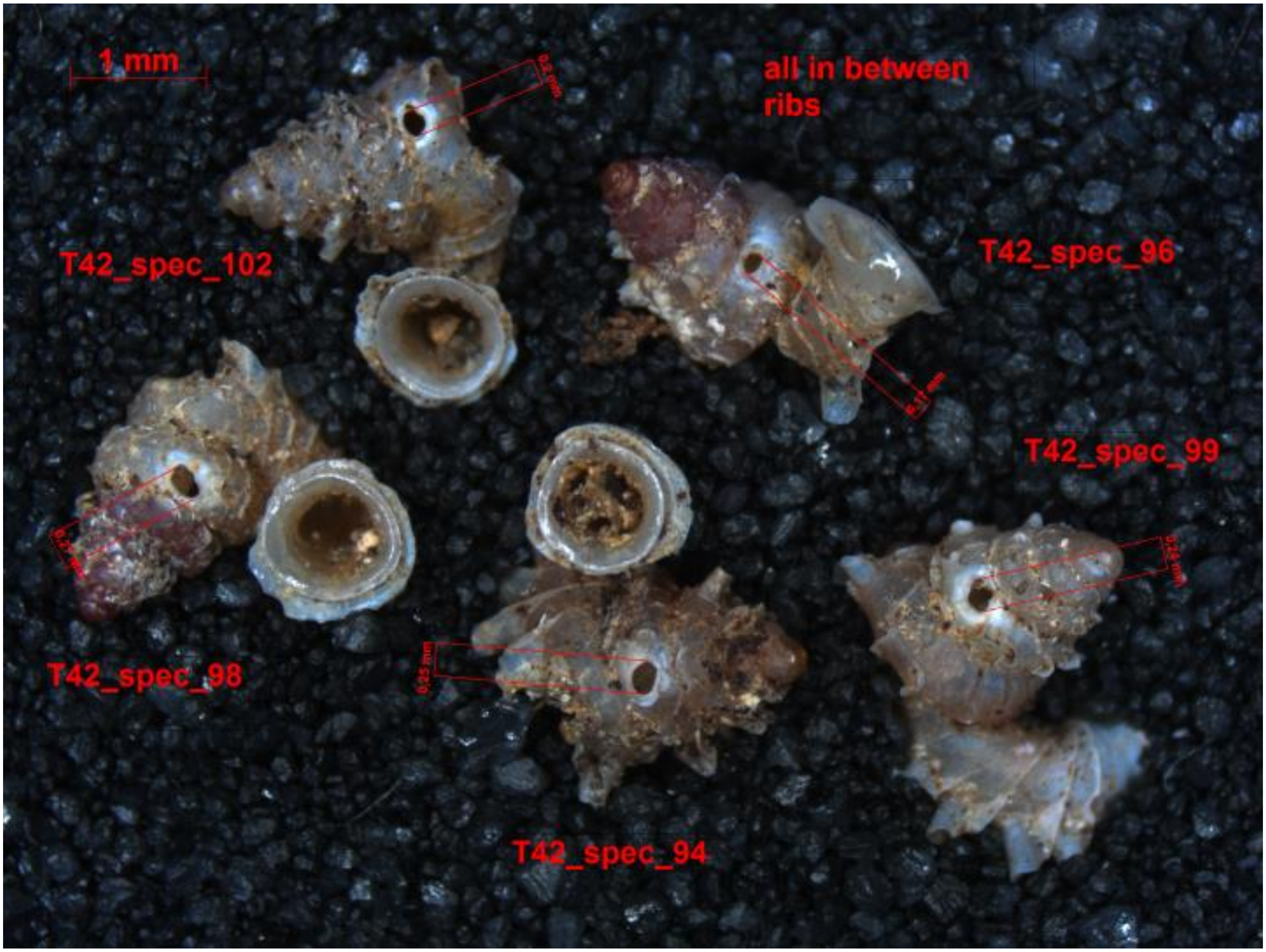

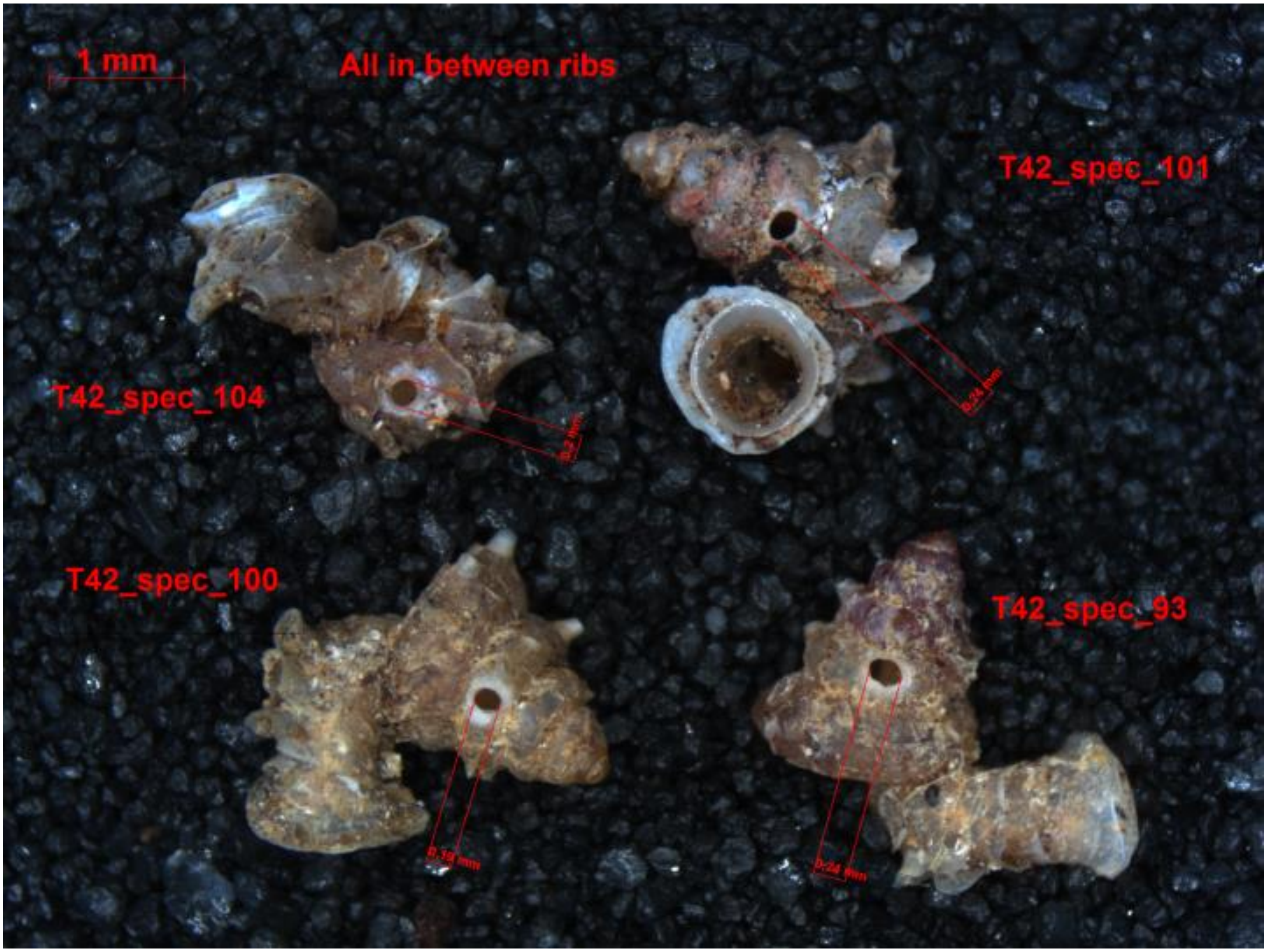
1 mm
All in between ribs
T42_spec_101
T42_spec_104
T42_spec_100
T42_spec_93

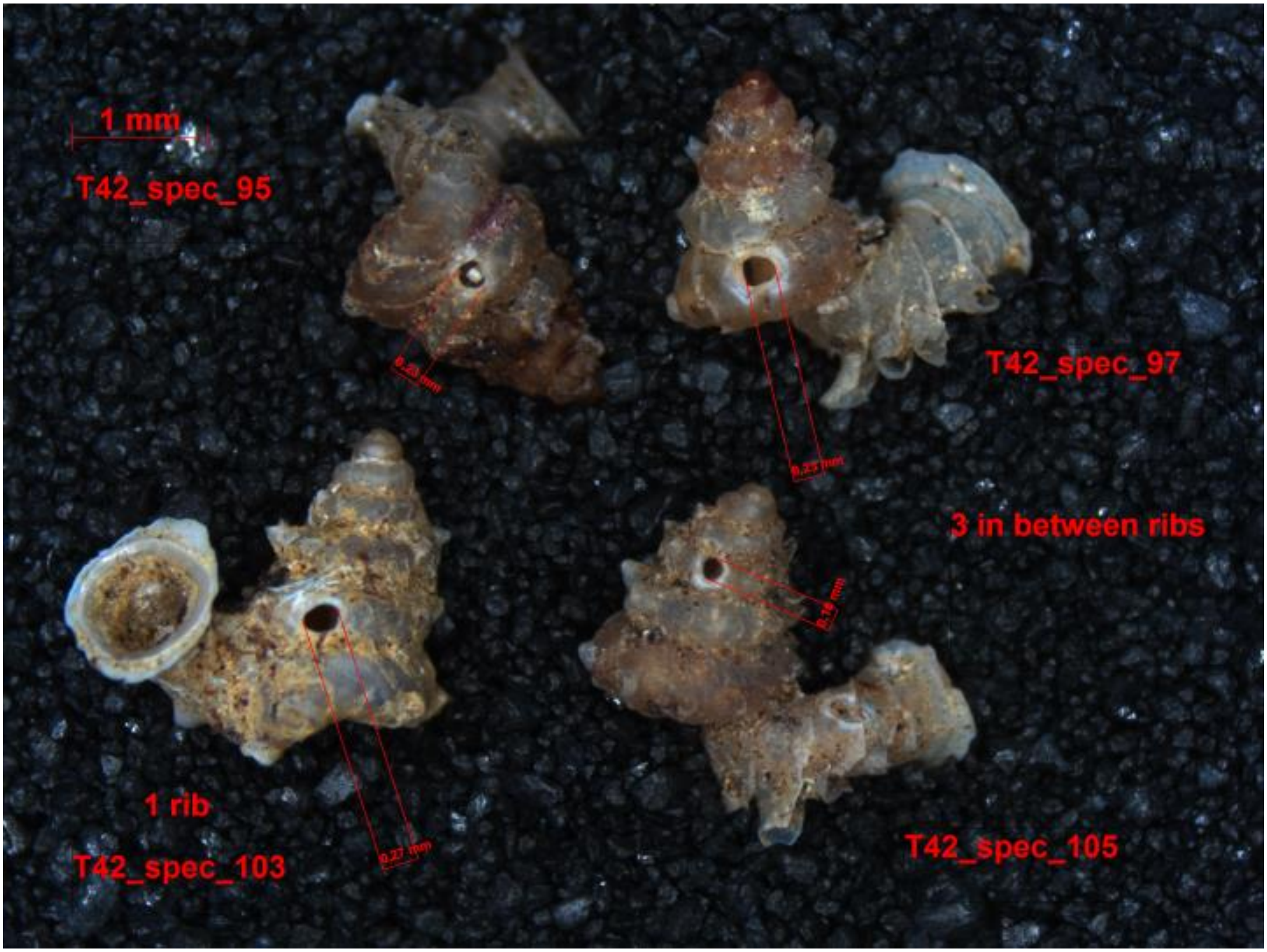

Figure S11. Specimens from population T 44.

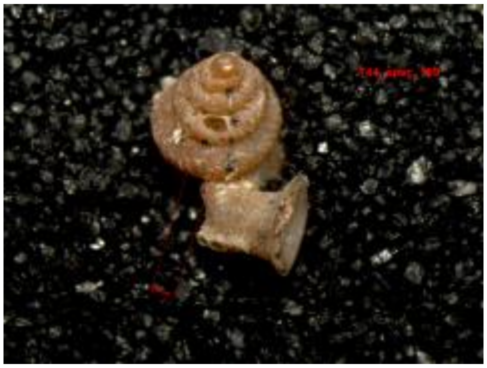

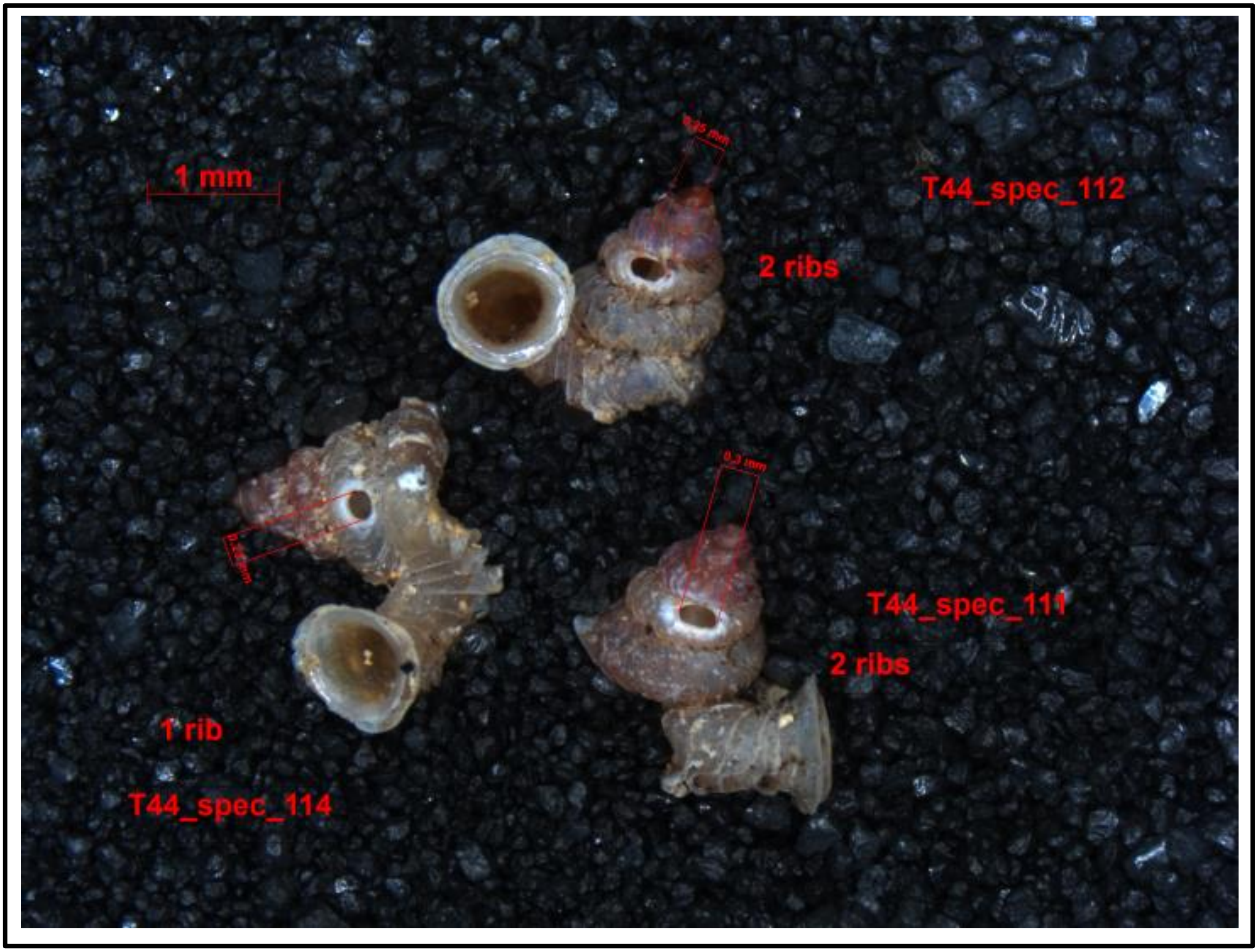
1 mm
T44_spec_112
2 ribs
T44_spec_111
2 ribs
1 rib
T44_spec_114

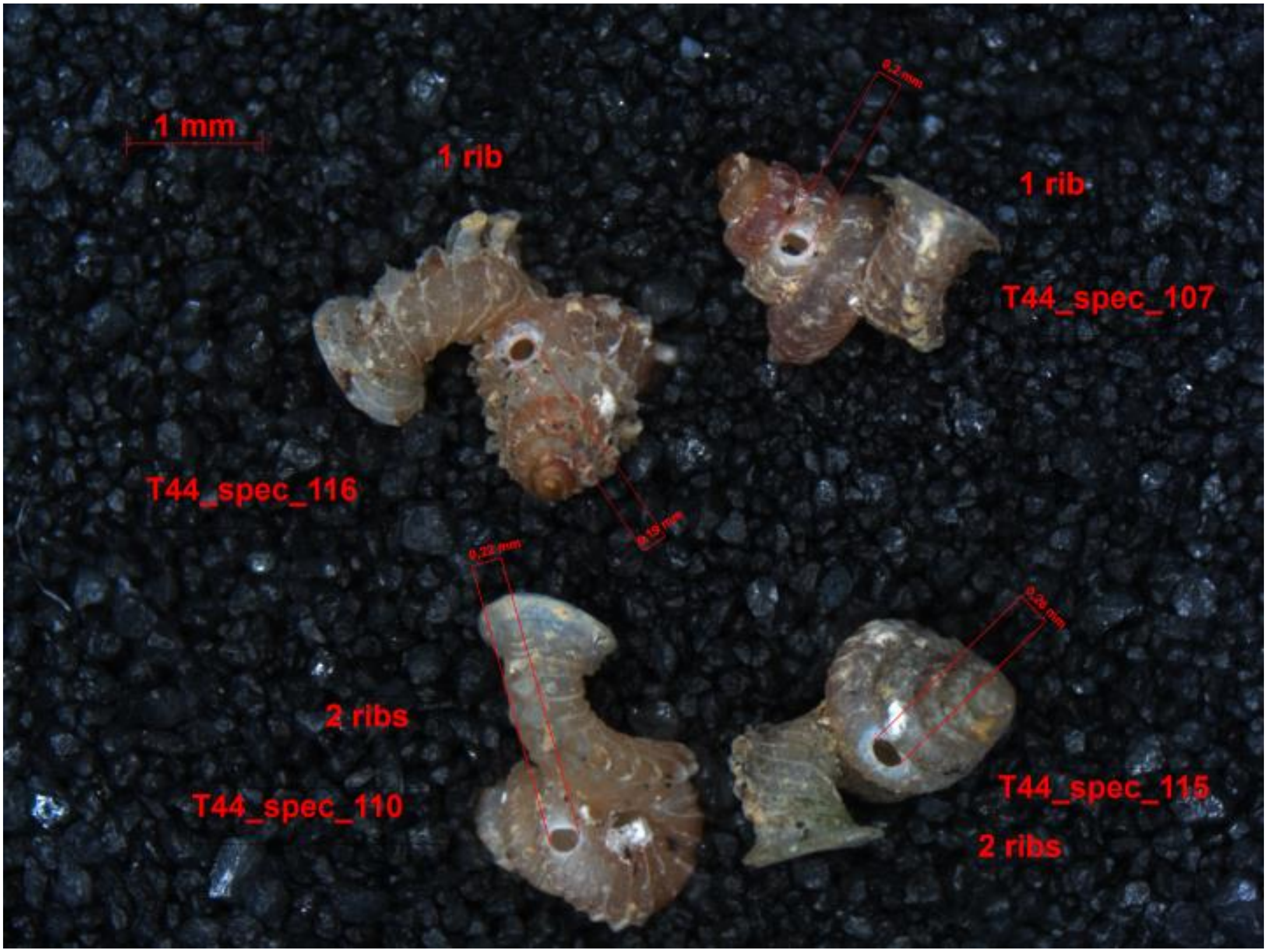
1 mm
1 rib
1 rib
T44_spec_107
T44_spec_116
2 ribs
T44_spec_110
T44_spec_115
2 ribs

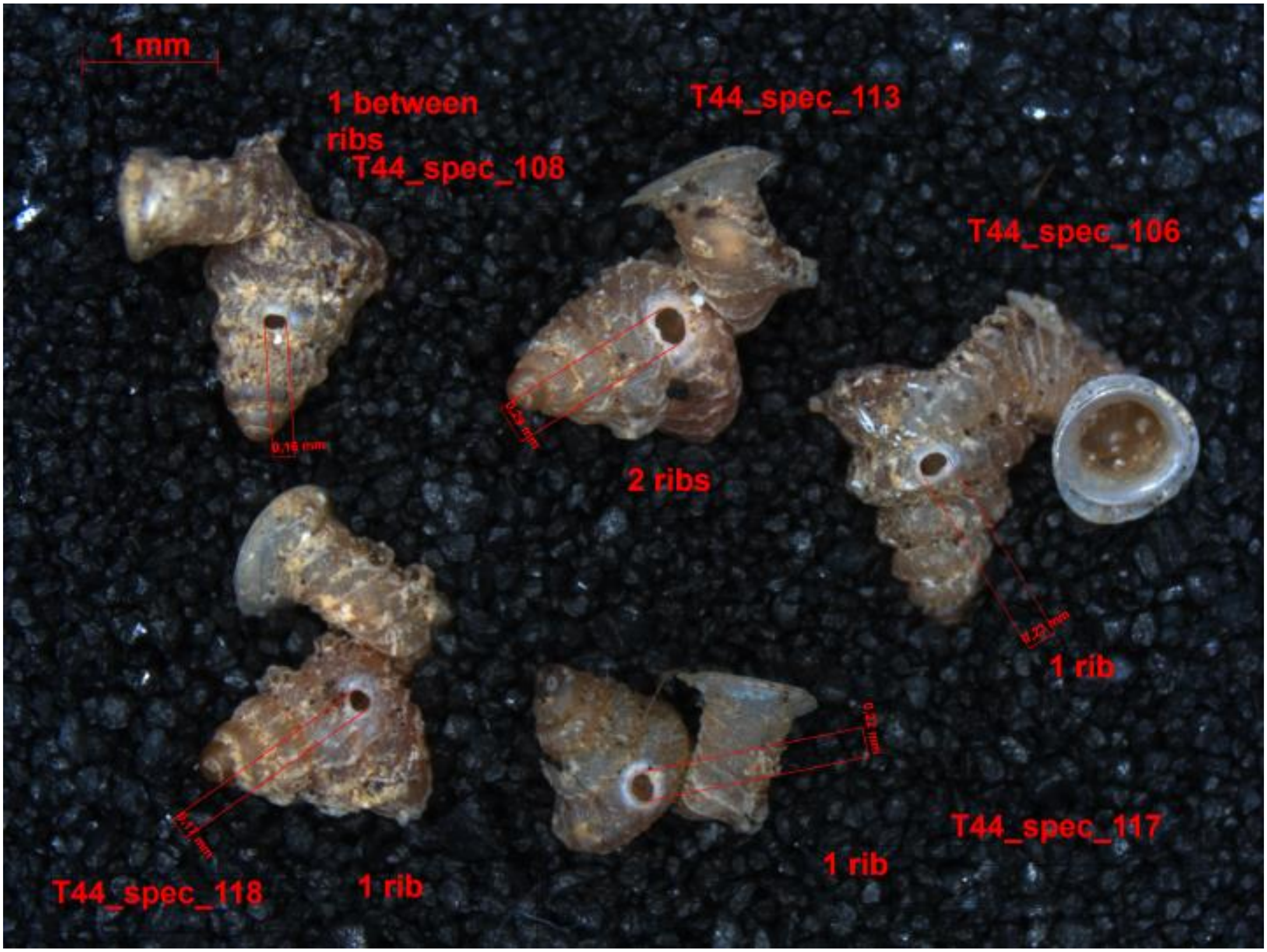
1 mm
1 between
ribs
T44_spec_108
T44_spec_113
T44_spec_106
2 ribs
1 rib
T44_spec_117
1 rib
T44_spec_118
1 rib

Figure S12. Specimens from population T 34.

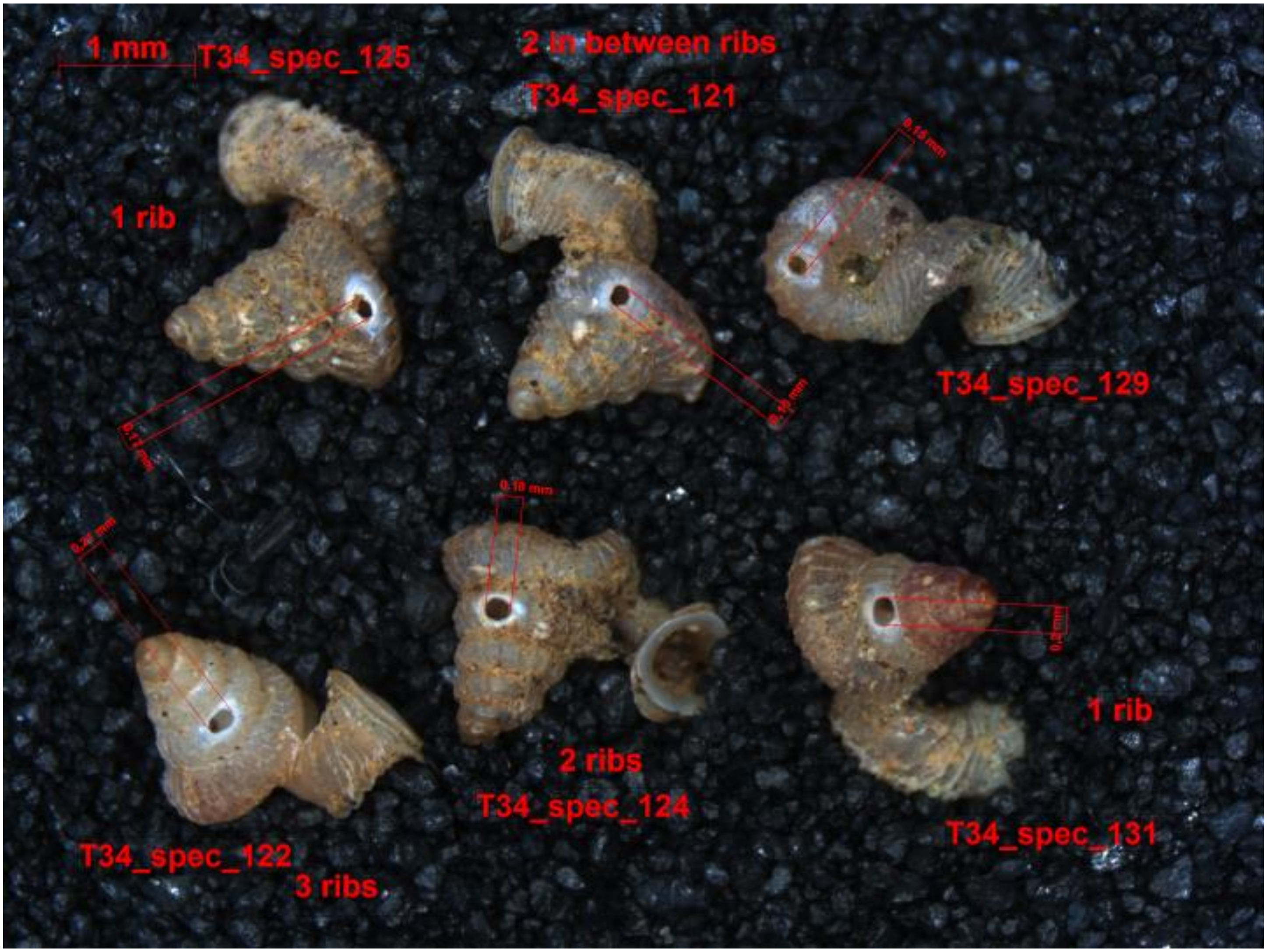

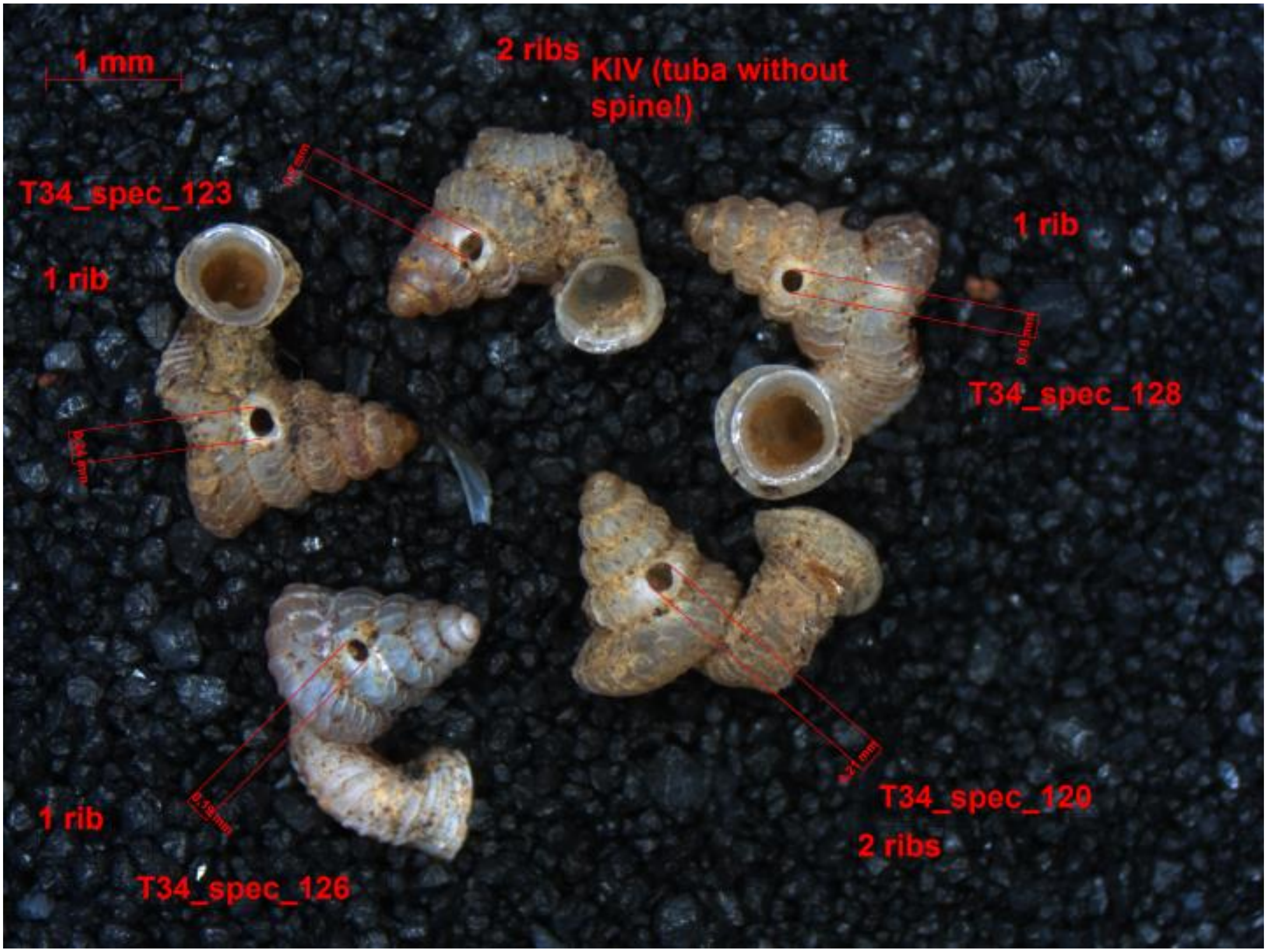
1 mm
2 ribs
KIV (tuba without spine!)
T34_spec_123
1 rib
1 rib
T34_spec_128
1 rib
T34_spec_120
2 ribs
T34_spec_126

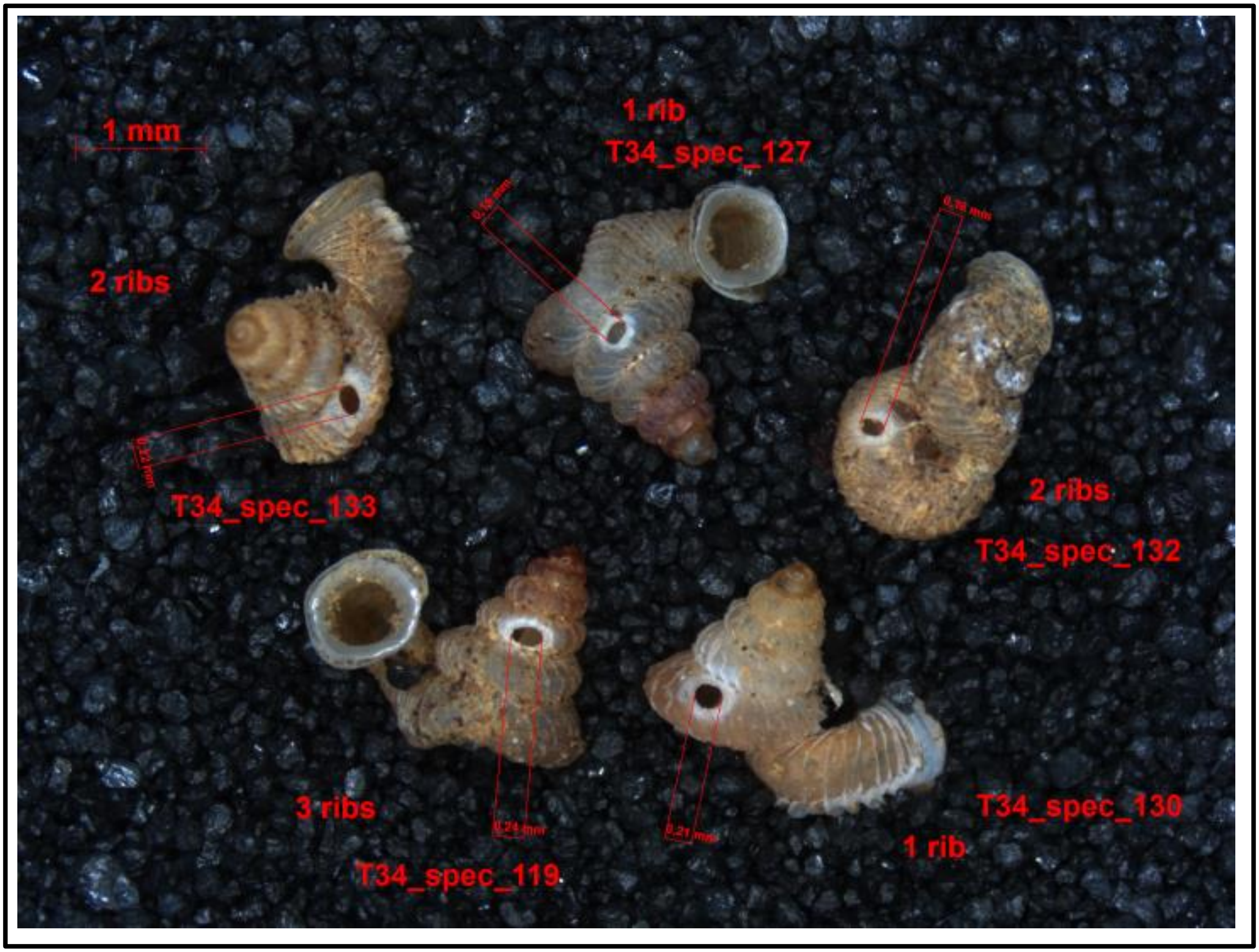
1 mm
1 rib
T34_spec_127
2 ribs
T34_spec_133
2 ribs
T34_spec_132
3 ribs
T34_spec_119
T34_spec_130
1 rib

Figure S13.Shells with two drill holes.

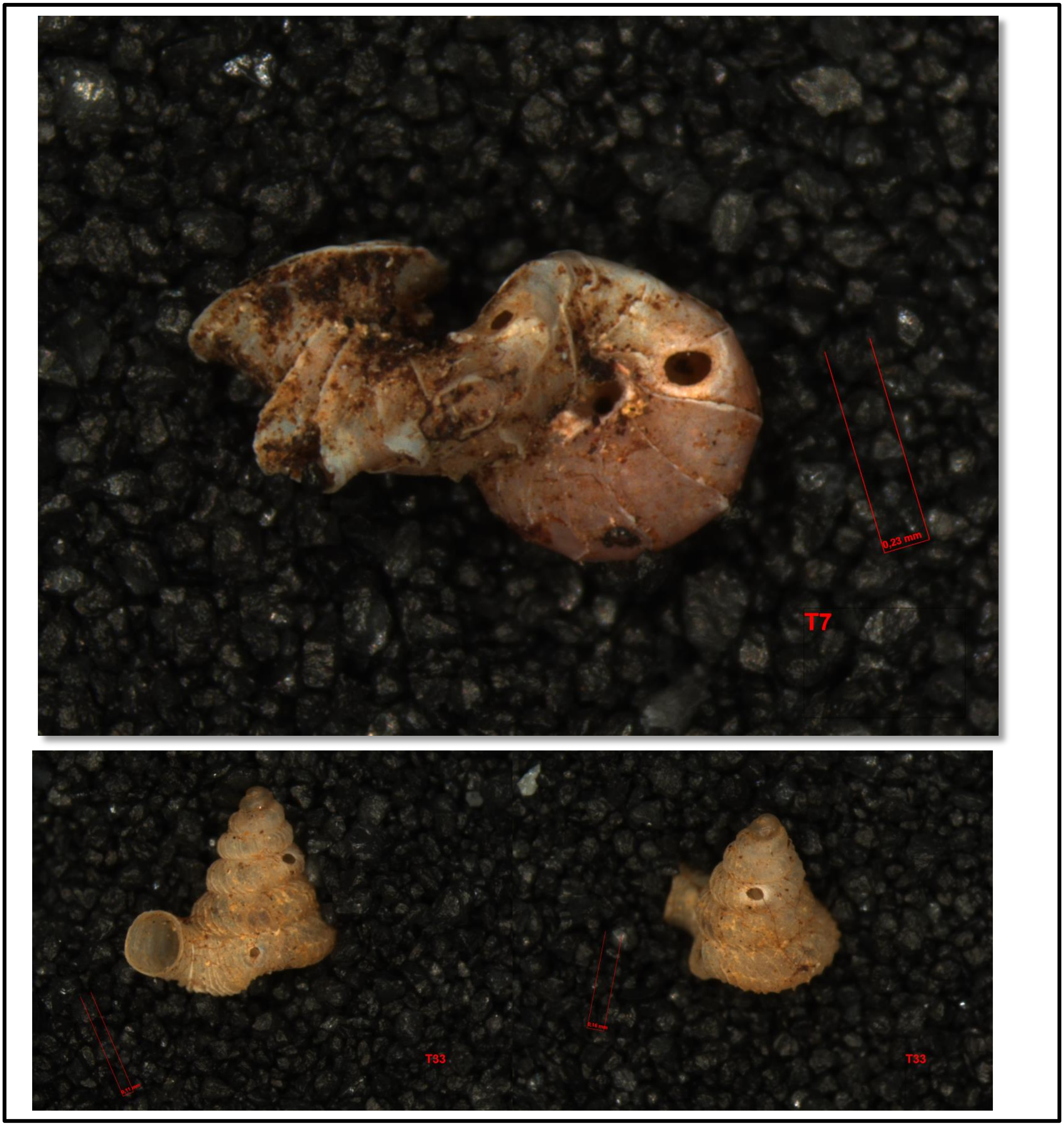

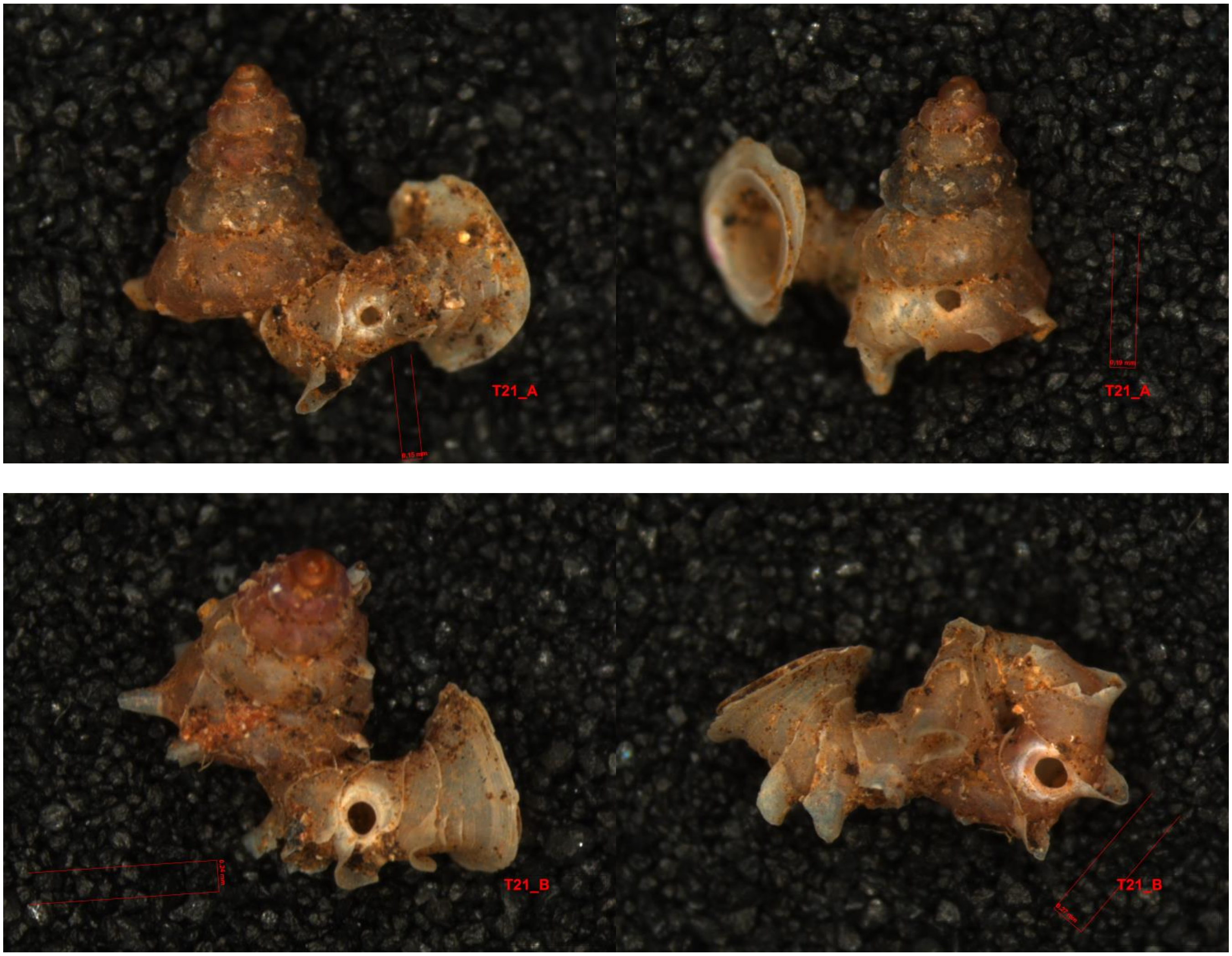
T21_A
T21_A
T21_B
T21_B

<u>Test 1 (c & d) - Correlation between ribs density and ribs intensity of *Plectostoma* shell.</u>

Table S2. Shell parameters for the 14 specimens in Test 1 (c).

Specimens	Total shell materials volume (shell whorl and ribs) (mm3)	Ratio between shell material of shell ribs to shell material of shell whorls	Number of ribs on shell	Shell materials volume (shell whorl only excluding ribs) (mm3)	Shell materials volume (shell ribs only) (mm3)	Shell thickness (mm)	Number of shell's spire whorls	Shell's spire height (mm)	Shell's spire width (mm)
Tomanggong Besar 21 C	1.02	0.25	47	0.82	0.21	0.04	5	2.05	1.5
Tomanggong Besar 22 A	0.96	0.22	48	0.79	0.17	0.04	4.75	2	1.6
Tomanggong Besar 21 D	1.02	0.24	50	0.82	0.2	0.04	4.88	2.15	1.6
Tomanggong Besar 21 A	1.01	0.18	54	0.86	0.15	0.05	5.13	2.05	1.5
Tomanggong Besar 44 B	0.65	0.16	72	0.56	0.09	0.03	5.25	2.2	1.5
Tomanggong Besar 42 C	0.82	0.17	77	0.7	0.12	0.03	5.38	2.2	1.5
Tomanggong Besar 7 C	0.87	0.19	77	0.73	0.14	0.04	5.38	2.1	1.5
Tomanggong Besar 42 A	0.82	0.19	78	0.69	0.13	0.04	5.25	2.1	1.5
BOR 2991 A	0.69	0.12	87	0.61	0.07	0.03	5.5	2.05	1.55
BOR 2991 C	0.7	0.11	93	0.63	0.07	0.04	5.63	2.05	1.6
BOR 2991 B	0.71	0.11	111	0.63	0.07	0.04	5.75	2.3	1.65
Tomanggong Besar 33 B	0.37	0.02	121	0.36	0.01	0.03	4.86	1.3	1.65
Tomanggong Besar 33 A	0.37	0.03	129	0.36	0.01	0.03	4.63	1.3	1.5
Tomanggong Besar 33 C	0.37	0.02	138	0.36	0.01	0.03	4.75	1.3	1.55

Table S3. Spearman correlations between anti-predatory shell traits and shell size of *Plectostoma* snails (n = 14). There are no statiscal signicant correlations were found. All analyses were done with Pearson correlation except the bolded text that were done with Spearman correlation.

		SHELL SIZE MORPHOMETRICS			
		**Number of shell's spire whorls**	**Shell's spire height (mm)**	**Shell's spire width (mm)**	**Shell materials volume (shell whorl only excluding ribs) (mm^3)**
**ANTI-PREDATORY SHELL TRAITS' MORPHOMETRICS**	**Number of ribs on shell**	-0.07 n.s.	**-0.34** n.s.	**0.28** n.s.	-0.92***
	**Shell materials volume (shell ribs only) (mm3)**	0.05 n.s.	**0.36** n.s.	**-0.31** n.s.	0.95***
	**Shell thickness (mm)**	0.08 n.s.	**0.17** n.s.	**-0.12** n.s.	0.81***

n.s. Not statistically significant correlation.

*** $p < 0.001$.

Figure S14. Shell figures of the 14 specimens in Test 1 (c).

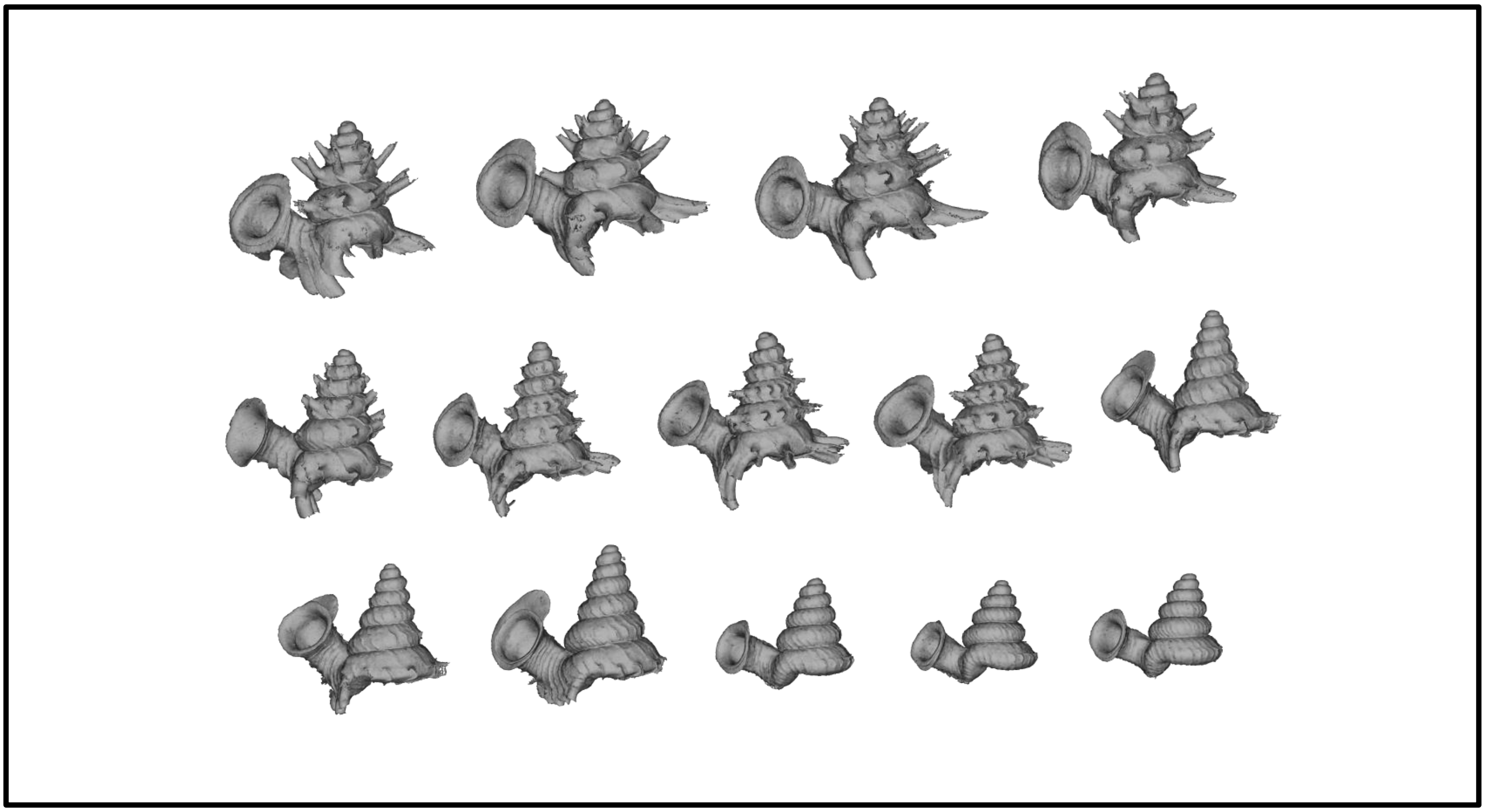

Figure S15. A graph shows the correlation between total shell materials and number of ribs.

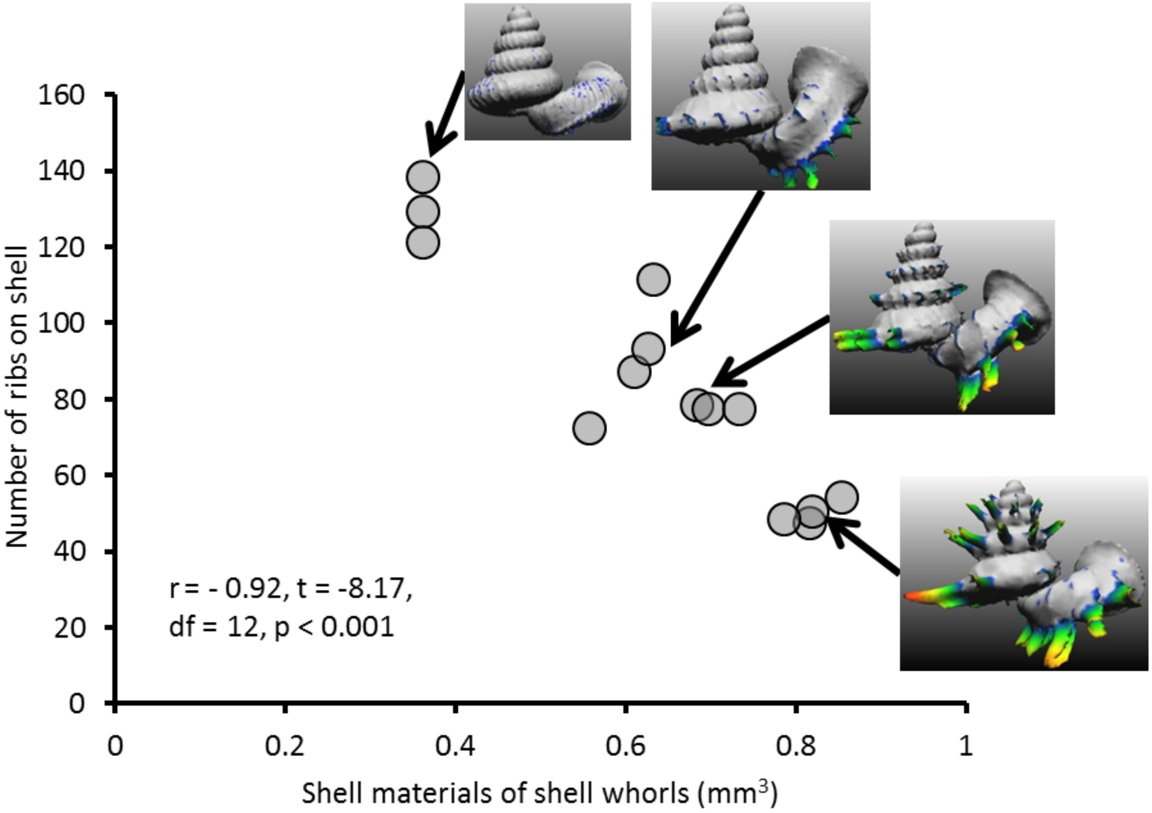

Figure S16. A graph shows the correlation between total shell materials and ribs intensity.

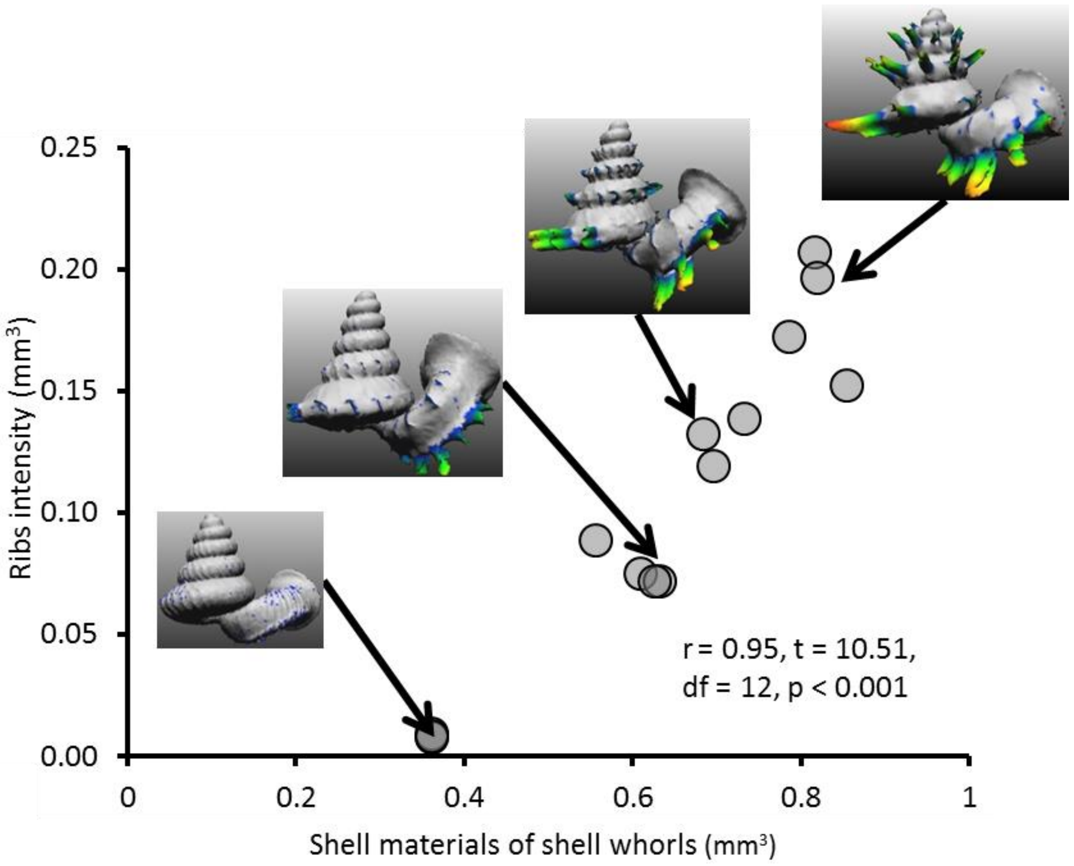

Figure S17. A graph shows the correlation between total shell materials and shell thickness.

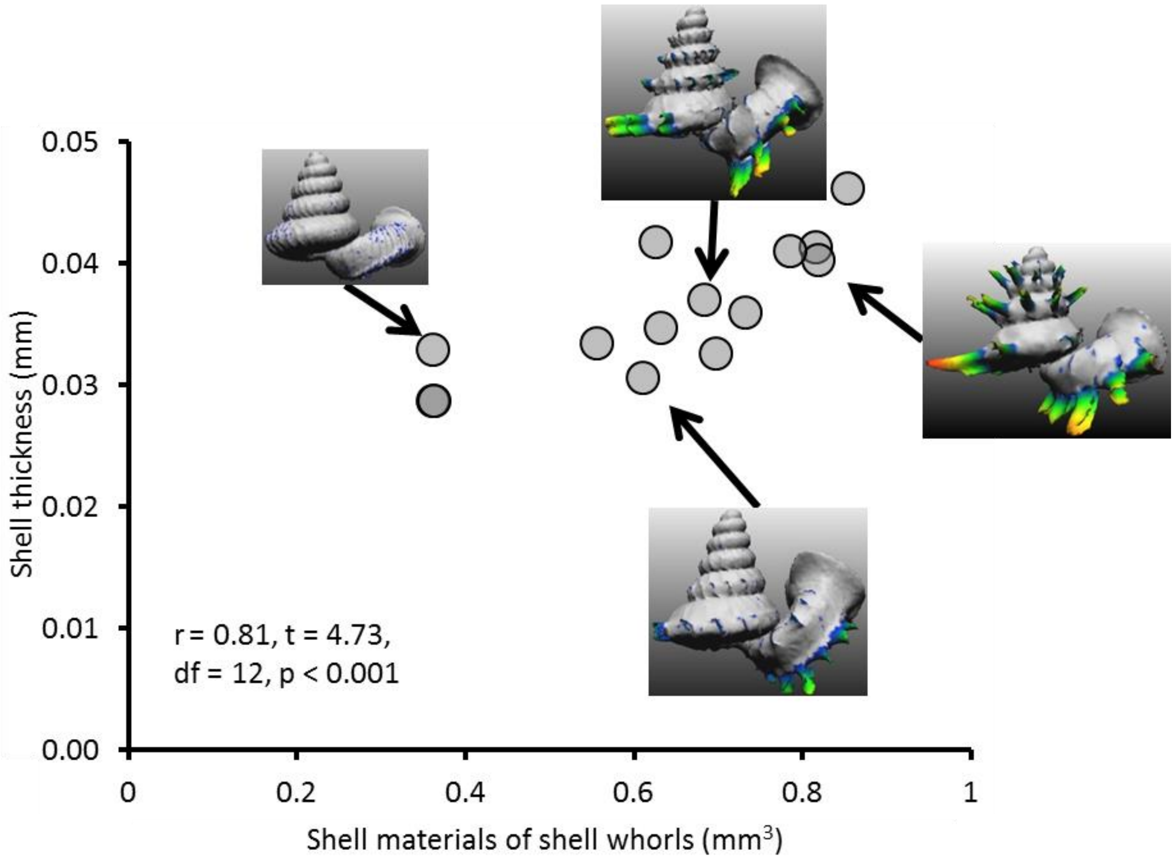

Test 2 (a) – Predator's predatory behaviour vs. prey's tuba

Table S3. Experiment data of Test 2 (a).

No.	Slug	Experiment starting time	Time when attacking started	Duration	adult	Sub-adult	juvenile
1	S1	22:04, 18/01/2013	14:00 - 18:30, 19/01/2013	4:30	0	1	1
3	S2	11:50, 20/01/2013	22:00, 20/01 - 06:00, 21/01	8:00	0	1	0
5	S2	06:30, 21/01/2013	13:00, 21/01 - 22:20:00, 21/01	9:20	0	1	1
7	S2	22:22, 21/01/2013	22:22, 21/01/2013 - 06:45, 22/01/2013	9:07	0	1	1
8	S2	06:45, 22/01/2013	21:50, 22/01/2013 - 05:30, 23/01/2013	9:20	0	1	1
9	S2	05:30, 23/01/2013	15:00 - 18:00, 23/01/2013	3:00	0	1	Missing
10	S2	18:15, 23/01/2013	18:15, 23/01/2013- 10:55, 24/01/2013	16:40	0	1	1
11	S2	11:00, 24/01/2013	18:15, 24/01/2013- 09:00, 25/01/2013	14:45	0	1	0
12	S2	09:00, 25/01/2013	23:00, 25/01/2013 - 06:00, 25/01/2013	7:00	0	1	1

Figure S18. Shell specimens used in Test 2 (a). Green box – survival specimen, red box – specimen that was eaten by *Atopos* slug. Yellow circle - shellwith intact operculum.

Experiment No. 1.

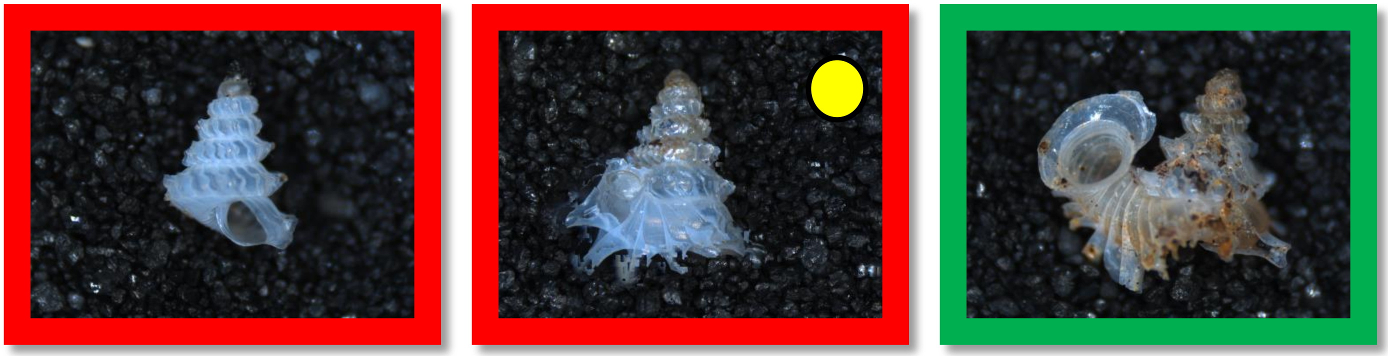

Experiment No. 3.

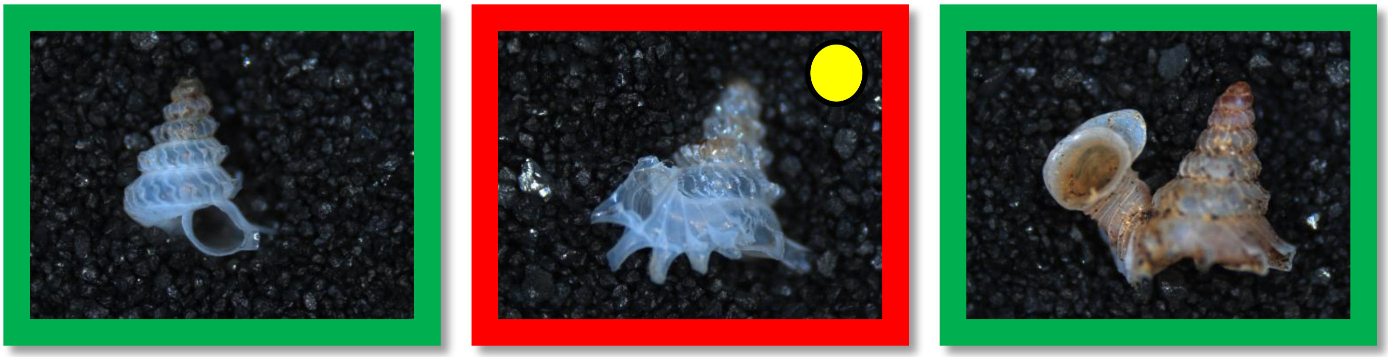

Experiment No. 5.

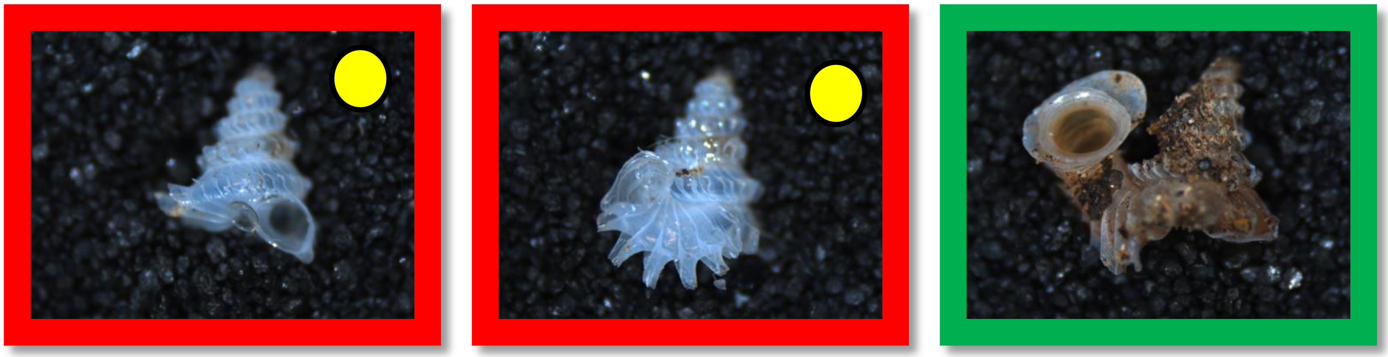

Experiment No. 7.

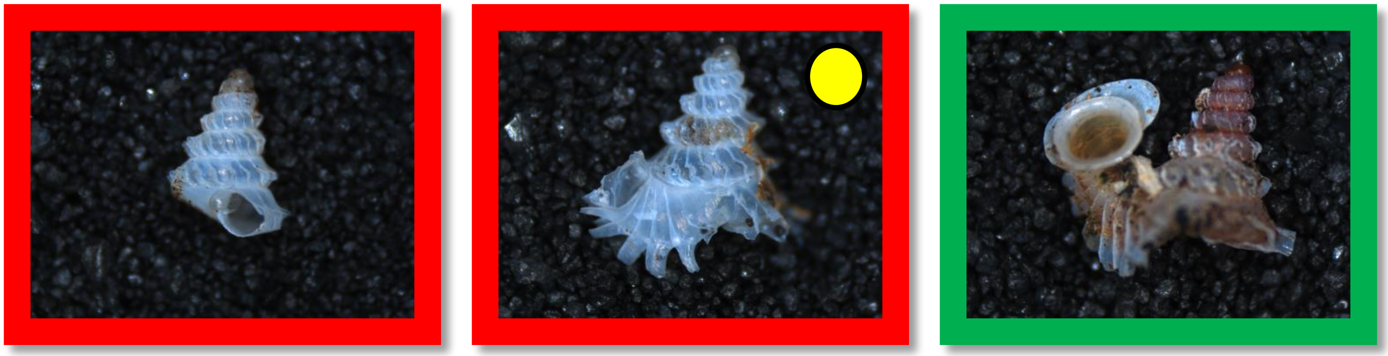

Experiment No. 8.

Specimen waslost before photograph could be taken.

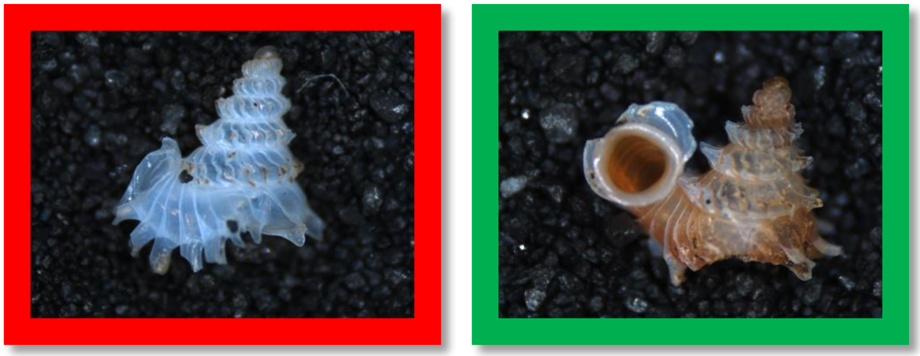

Experiment No. 9.

Specimen cannot be located after experiment

?

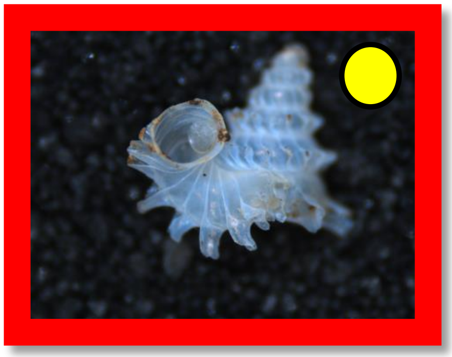

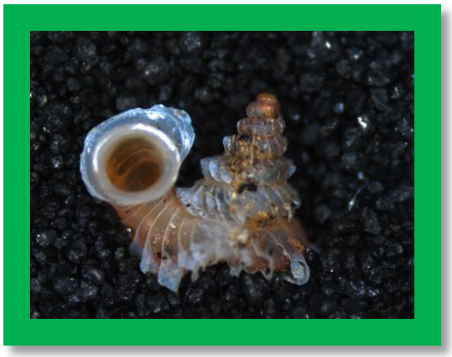

Experiment No. 10.

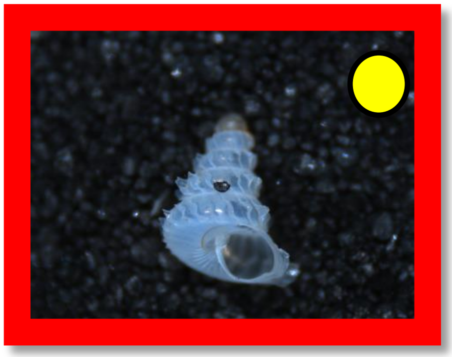

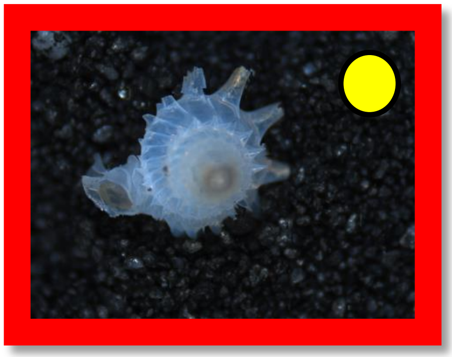

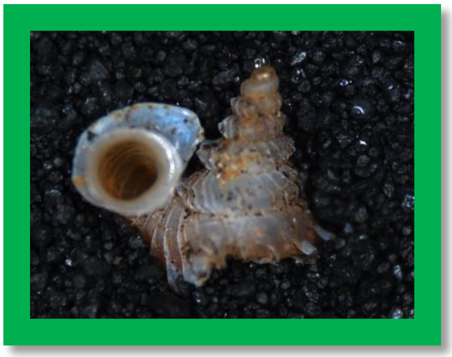

Experiment No. 11.

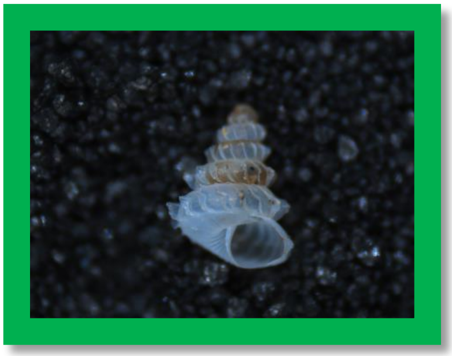

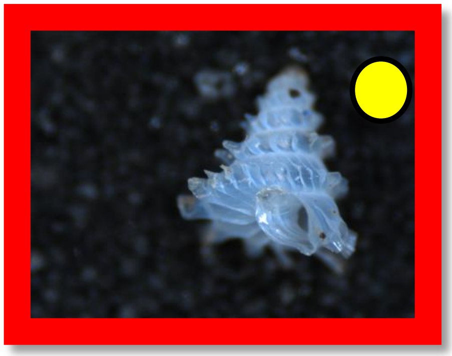

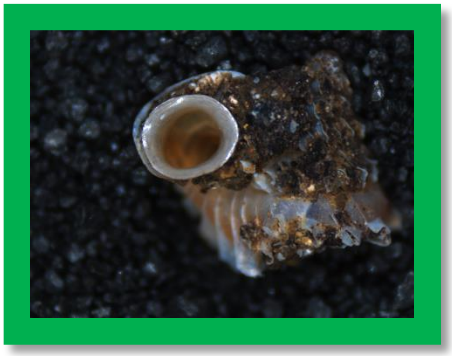

Experiment No. 12.

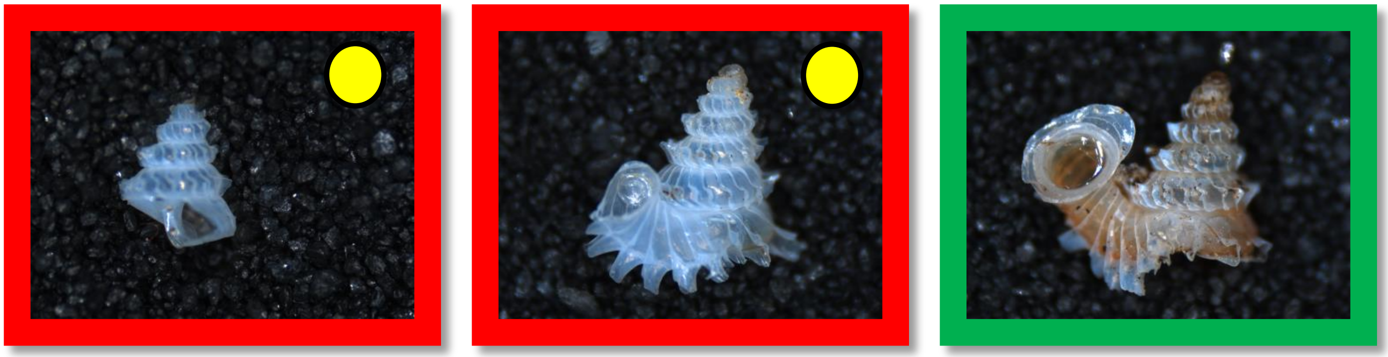

Test 2 (b) – Association between slug's shell- apertural entry behaviour, snail anti-predatory behaviour.

Figure S19. Frequency of the drill hole locations of the of 133 shells.

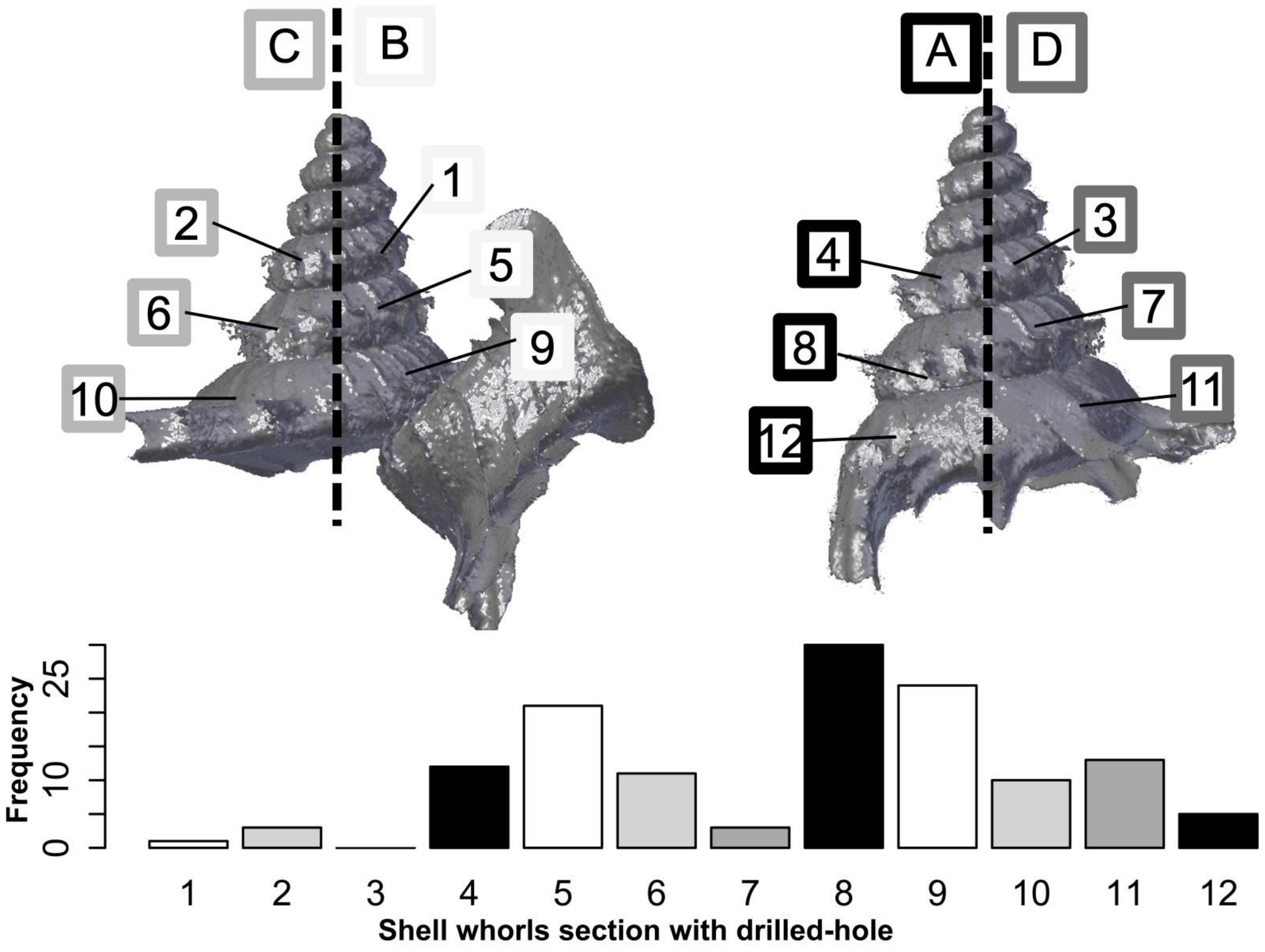

Test 2 (c) – Association between slug's shell- apertural entry behaviour, snail anti-predatory shell's traits.

Figure S20. Radius of curvature changes along ontogeny axis of *Plectostoma concinnum* shell.

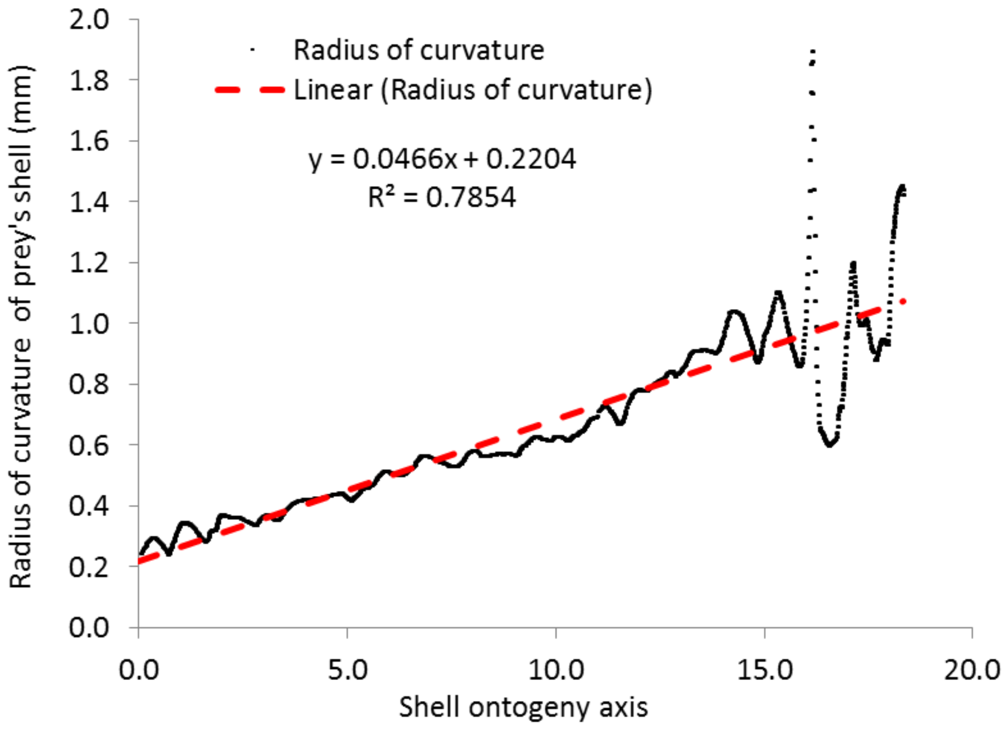

Figure S21. Predatory path distance changes along ontogeny axis of *Plectostoma concinnum* shell.

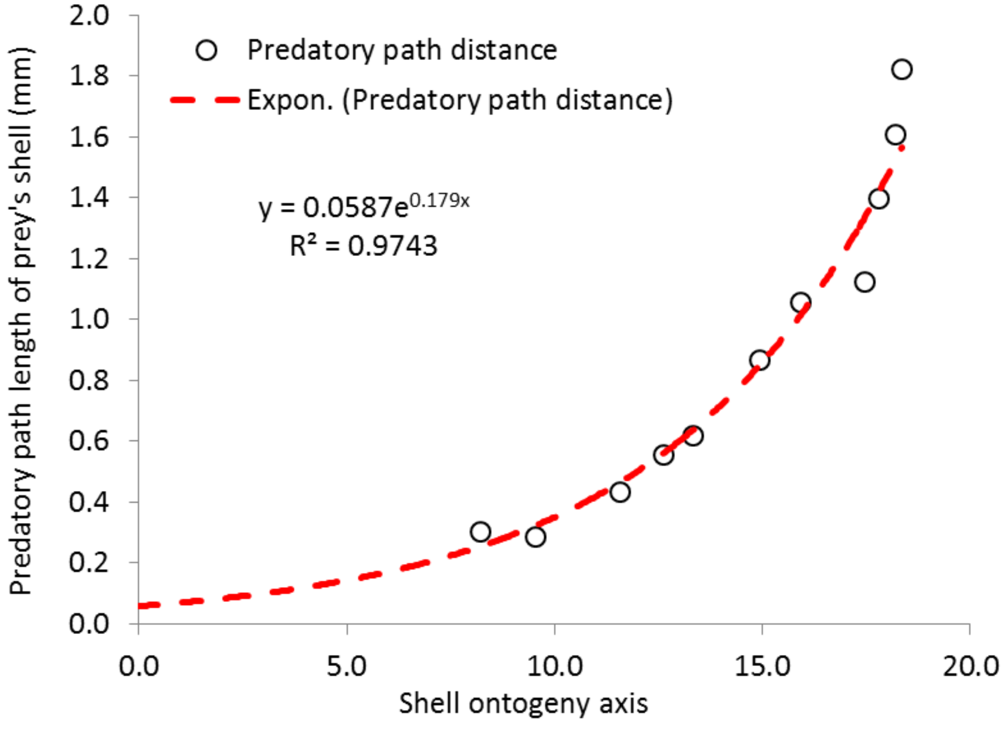

Figure S22. Torsion changes along ontogeny axis of *Plectostoma concinnum* shell.

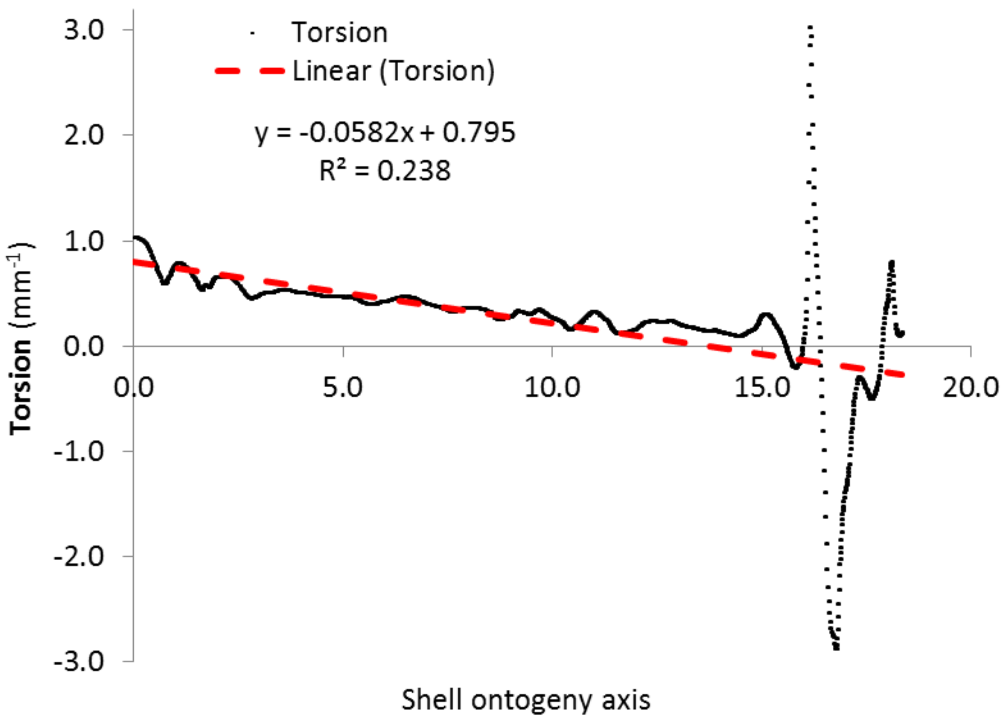

Torsion
Linear (Torsion)
y = -0.0582x + 0.795
R² = 0.238
3.0
2.0
1.0
0.0
-1.0
-2.0
-3.0
Torsion (mm-1)
0.0
5.0
10.0
15.0
20.0
Shell ontogeny axis

Figure S23. Examples of other *Plectostoma* species that were found in Peninsular Malaysia with drill hole that probably made by *Atopos* slug.

Plectostoma christae **(Maassen, 2001)**
V 9207

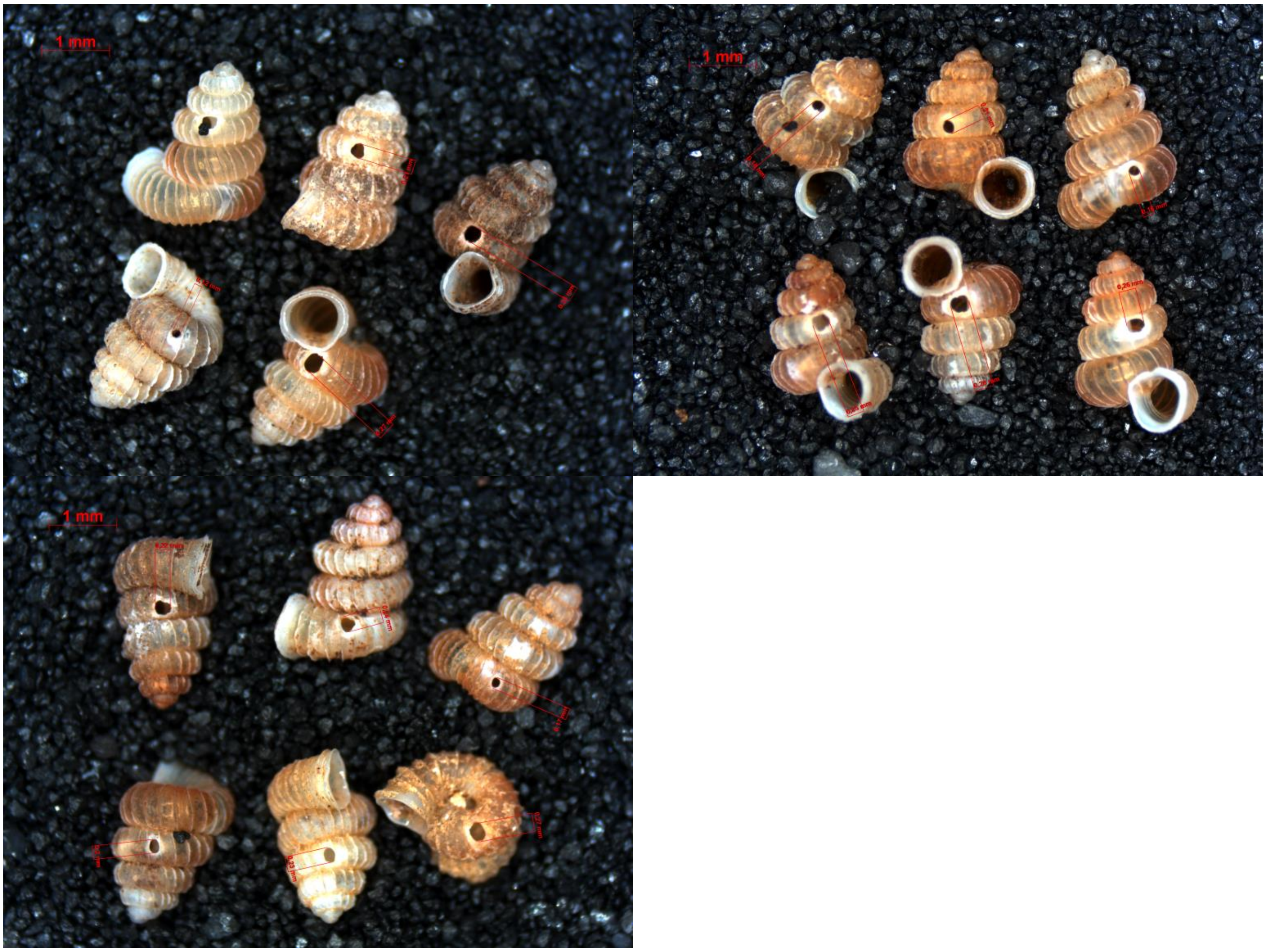

Plectostoma senex **(van Benthem Jutting, 1952)**
BBOR 5628

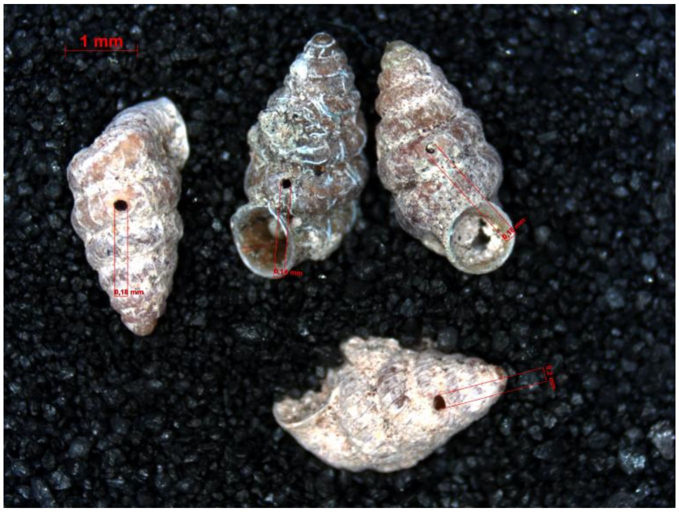

***Plectostoma ikanensis* Liew, Vermeulen & Schilthuizen 2014**
V 9446

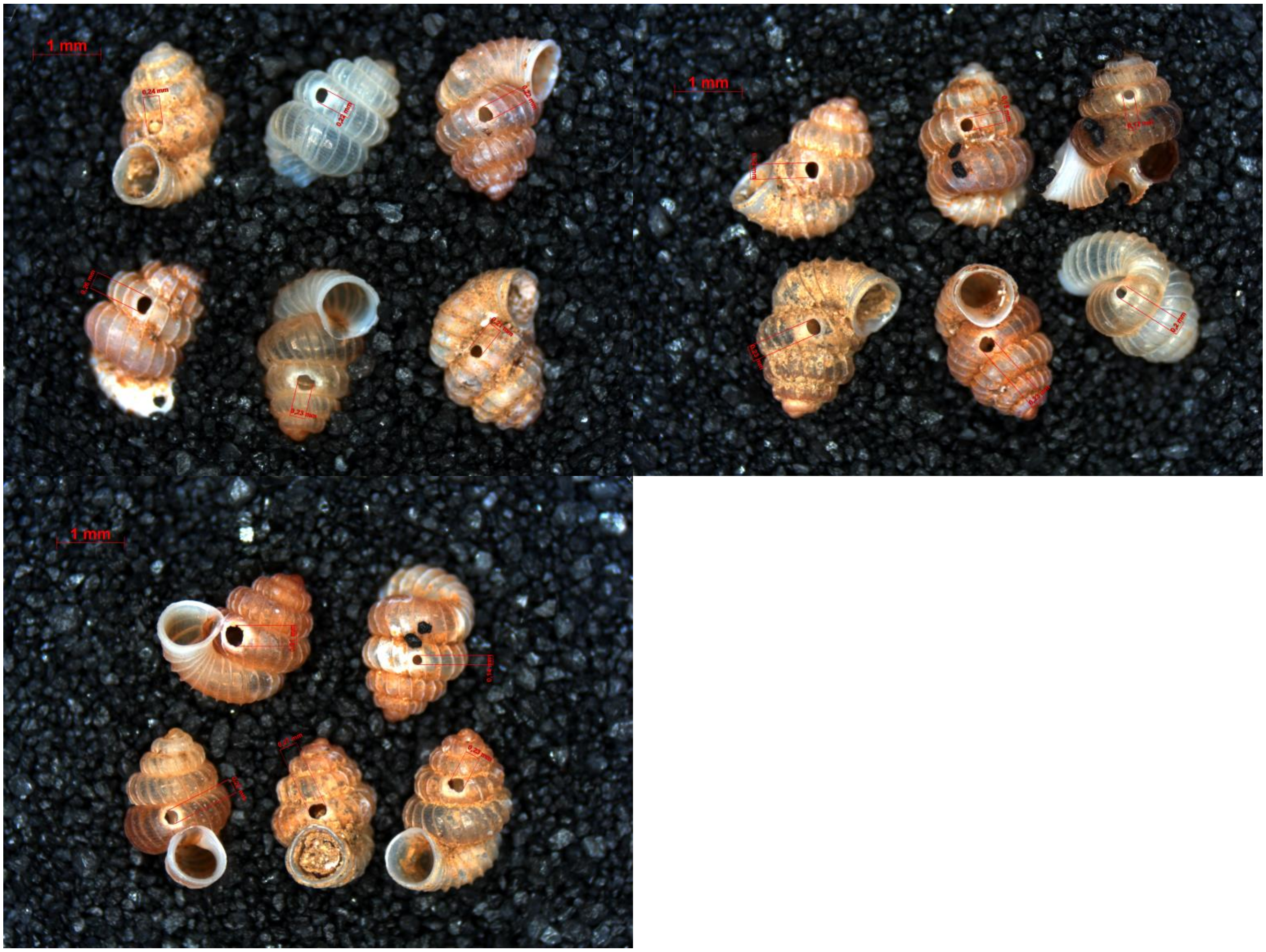

***Plectostoma siphonostomum* (van Benthem Jutting, 1952)**
V8199

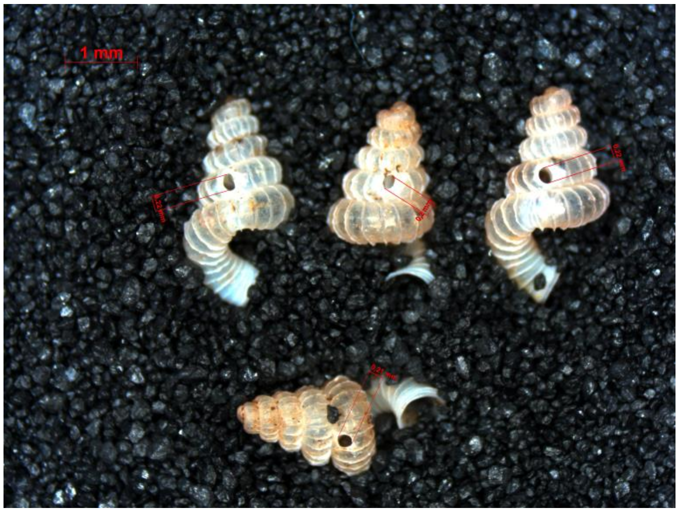
1 mm
